# Comparative transcriptome analysis reveals phytohormone signalings, heat shock module and ROS scavenger mediate the cold-tolerance of rubber tree

**DOI:** 10.1038/s41598-018-23094-y

**Published:** 2018-03-21

**Authors:** Xiaomin Deng, Jianxiao Wang, Yan Li, Shaohua Wu, Shuguang Yang, Jinquan Chao, Yueyi Chen, Shixin Zhang, Minjing Shi, Weimin Tian

**Affiliations:** 1 Ministry of Agriculture Key Laboratory of Biology and Genetic Resources of Rubber Tree/State Key Laboratory Breeding Base of Cultivation and Physiology for Tropical Crops, Rubber Research Institute, Chinese Academy of Tropical Agricultural Sciences, Danzhou, Hainan 571737 P.R. China; 2 College of Landscape and Ecological Engineering, Hebei University of Engineering, Handan, 056021 Hebei China

## Abstract

Two contrasting cold response rubber tree clones, the cold-resistant ‘93-114’ and cold-sensitive ‘Reken501’, were subject to a global transcriptome response assessing via high-throughput RNA-seq technique and comprehensive bioinformatics analysis using the referenced rubber tree genome with the purpose of exploring the potential molecular cues underlying the tolerance of rubber trees to cold stress. As a result, a total of 1919 genes had significantly higher expression, while 2929 genes had significantly lower expression in ‘93–114’ than in ‘Reken501’ without cold stress. Upon cold stress, the numbers of genes with significantly higher expression decreased to 1501 at 1 h treatment and to 1285 at 24 h treatment in ‘93–114’ than that of ‘Reken501’, conversely, the numbers of genes with significantly lower expression increased to 7567 at 1 h treatment and to 5482 at 24 h treatment. Functional annotation of the differentially expressed genes between ‘93–114’ and ‘Reken501’ suggests that down-regulation of auxin and ethylene signaling and activation of heat shock module and ROS scavengers is a primary strategy for *H. brasiliensis* to cope with cold stress. Our identified vital differentially expressed genes may be beneficial for elucidation of the molecular mechanisms underlying cold tolerance and for genetic improvement of *H. brasiliensis* clones.

## Introduction

Rubber tree (*Hevea brasiliensis* Muell. Arg.) is a primary source of natural rubber (NR) worldwide. The rubber tree originating in the Amazon rainforest has been commercially planted on a large scale in South or Southeast Asia as well as in tropical areas between 10 degrees latitude near the Earth’s equator, where so-called traditional rubber tree planting areas without cold stress are located. To meet the demand for NR, rubber tree planting has been continuously expanded and is no longer limited to traditional areas. For example, the rubber tree has been successfully grown in several areas of South China, where the tropical north fringe region and a sudden cold wave frequently occur. Many rubber cultivars introduced from the Amazon rainforest and Southeast Asia suddenly died following exposure to extreme cold stress in Hainan province. To cope with the cold damage to the rubber tree, artificial hybridization and selection of surviving rubber tree clones after cold stress were performed to develop rubber clones with relatively high potential cold tolerance, and several clones were obtained. The rubber tree clone ‘93–114’ was the hybrid offspring of the preliminary rubber tree parents clones ‘Tianren 31–45’ and ‘Hekou311’. The resistance of rubber tree clone ‘93–114’ to cold stress has been verified in the field for more than 40 years. Conversely, the rubber tree clone ‘Reken501’, the hybrid offspring of the parents rubber trees clones RRIM600 and PB260 introduced from Malaysia, is cold-sensitive.

The refined rubber tree genome sequence was recently reported^1,2^, serving as useful reference genome sources for gene expression analyses to reveal molecular mechanisms of cold stress in the rubber tree. RNA-seq has been widely applied to elucidate the response of plants to cold stress. The plant species include *Chorispora bungeana*^3^, *Lilium lancifolium*^4^, *Ammopiptanthus mongolicus*^5^, *Populus euphratica*^6^, Grapefruits^7^, *Vitis amurensis*^8^, rice^9^ and *Arabidopsis*^10^, as well as *Jatropha curcas*^11^, *Anthurium andraeanum*^12^ and *Elaeis guineensis*^13^. The available data show that the responses of different plant species to cold stress are both conserved and distinctive to some extent. Extensive studies have revealed that the jasmonate (JA) signaling module of COI1 (Coronitine insensitive 1) -JAZ (Jasmonate zim-domain) –ICE1 (Inducer of CBF expression) responds positively to cold stress and that overexpression of *ICE1* enhances the resistance of plants to cold stress by activating CBF (C-repeat binding factor) or DREB (dehydration responsive element-binding) factors^14,15^. In contrast, ethylene signaling can negatively regulate plant responses to cold stress partly by inhibiting the functions of CBF or DREB^16^. In addition, ABA (abscisic acid) dependent and ABA independent pathways in many plants can regulate the activities of DREB or CBFs during cold stress^17^, and thus CBF or DREB transcription factors function as crosstalk points among these three signaling pathways. ABA signaling activation under cold stress often occurs in temperate plants during cold acclimation, while the rubber tree, a tropical woody plant, often suffers from sudden cold stress without cold acclimation. Whether ABA signaling also plays a role in the response to cold stress to promote the survival of rubber tree clone ‘93–114’ has remained unclear to date.

Plant heat shock modules composed of heat shock protein (HSP) and heat shock factor (Hsf). Although Hsfs and HSPs are first described to function under high temperature conditions, both of them have also been shown to participate in cellular responses induced by high and low temperatures, which has raised extensive attention toward elucidating their role in stress responses both in woody and non-woody plants^8,18–22^. Moreover, an intense cross-talk between heat and cold stress regulatory networks mediated by HSFs and HSPs in *Arabidopsis* is proposed by Swindell *et al*. (2007)^23^ and the highly parallel signaling pathways in low and high temperature are presented by Catalá *et al*. (2012) as well^24^, suggesting that plants may retain similar molecular mechanism in responses to external temperature stresses^24,25^. In *Arabidopsis*, the expression of *AtHsfA3* is directly regulated by AtDREBA2 during heat stress response^26^, whether AtDREB2-AtHsfA3-AtHSPs modules are also involved in cold stress responses is remained elucidation. Upon cold stress, the DREB1-type/CBFs, Hsfs and HSPs genes are induced in many plants, e.g. *Arabidopsis*^23^, *Populus euphratica*^27^, and a cold-tolerant Chinese wild *Vitis amurensis*^8^, etc. Notably, overexpression of *Arabidopsis AtHsf1b* or tomato *CPsHSP* or DnaJ/HSP40/LeCDJ1 enhances chilling tolerance in transgenic tomato^28–30^. In addition, extreme temperature stresses induced wheat *TaHsf3* gene enhances transgenic *Arabidopsis* tolerance to cold and heat stresses when overexpressed^31^. These findings are clearly indicated that Hsf-type transcriptional factor and HSP proteins can indeed increase plants’ tolerance to external cold stress via transgenic technique. Importantly, Hsf-HSP modules can activate and retain the activities of reactive oxygen species (ROS) scavengers to protect plants against oxidative damage during abiotic stresses^32,33^. Our previous physiological study has revealed that the cold tolerance of rubber tree clone ‘93–114’ may be associated with the pulsed production of endogenous hydrogen peroxide and the enhanced activation of enzymatic and non-enzymatic ROS scavengers in response to cold stress when compared with the cold-sensitive rubber tree clone ‘Reken501’^34^. Therefore, physiological responses indicate that the expression of several genes encoding enzymatic and non-enzymatic ROS scavengers, such as HSP chaperone and ROS scavenger enzymes, may be enhanced in the cold-tolerant rubber tree clone ‘93–114’.

To reveal the molecular mechanism responsible for cold stress in the cold-tolerant rubber tree clone ‘93–114’, a comparative analysis of the comprehensive transcriptome responses to old stress of the cold-tolerant rubber tree clone ‘93–114’ and cold-sensitive rubber tree clone ‘Reken501’ was performed using the RNA-seq technique. The results will contribute to elucidating the strategies developed by the rubber tree to survive in non-traditional planting areas and further identify cold resistance-related genes for genetic improvement of *Hevea* cold tolerance.

## Results

### Illumina sequencing and data processing

The six distinct cDNA libraries from the two rubber tree clones (A0, A1 and A2, respectively, represent the cold-susceptible rubber clone without or with cold treatment for 1 h and 24 h; B0, B1and B2, respectively, represent the cold-resistance rubber clone without or with cold treatment for 1 h and 24 h) were separately sequenced using an Illumina HiSeq^TM^ 2000 RNA-seq platform, and approximately 8 Gbp raw reads were generated from each sample. The RNA-Seq data were first analyzed, and potential contamination by fungi and other foreign organisms was eliminated according to the stringent standard procedure of the Beijing Genomics Institute (BGI; Shenzhen, China). In addition to the careful sequencing analysis, raw reads containing adapter sequences and low quality reads were filtered and removed, producing 74.89 Mb (A0), 74.82 Mb (A1), 74.74 Mb (A2), 74.12 Mb (B0), 74.15 Mb (B1), and 74.39 Mb (B2) of clean reads (Table 1) (NCBI accession numbers: GSE67559). These high-quality clean reads were then mapped to the reference genome use HISAT^35^ (Hierarchical indexing for spliced alignment of transcripts). On average, 89.87% of the reads were mapped onto the rubber tree genome for each sample, suggesting that the samples were comparable. By using the annotation of gene information (Known genes) and the present sequenced transcriptome data (Novel genes), we finally identified 31360 (A0 sample), 31287 (A1 sample), 32107 (A2 sample), 31434 (B0 sample), 30924 (B1 sample), and 31044 (B2 sample) genes (Table 2) and measured their expression.

**Table 1:** Summary of sequencing reads after filtering.

Sample	Total Raw Reads (Mb)	Total Clean Reads (Mb)	Total Clean Bases (Gb)	Total Mapping Ratio
A0	80.37	74.89	6.74	89.73%
A1	80.37	74.82	6.73	90.1 3%
A2	80.37	74.74	6.73	90.1 8%
B0	82.96	74.1 2	6.67	89.75%
B1	82.96	74.1 5	6.67	89.99%
B2	82.96	74.39	6.70	89.44%

**Table 2:** Summary of expressed genes.

Sample	Total Clean Reads	Total Gene Mapping Ratio	Total Gene Number	Known Gene Number	Novel Gene Number	Total Transcript Number	Known TranscriptNumber	Novel TranscriptNumber
A0	74.89	72.60	31360	28258	3102	48730	23489	25241
A1	74.82	73.56	31287	28206	3081	48679	23477	25202
A2	74.74	72.38	32107	28994	3113	49816	24240	25576
B0	74.1 2	71.06	31434	28371	3063	49017	23689	25328
B1	74.1 5	73.85	30924	27947	2977	47735	23295	24440
B2	74.39	71.92	31 044	28019	3025	47629	23052	24577

### Differential gene expression among contrasting rubber tree cultivars

The differentially expressed genes (DEGs) in response to cold stress in the two contrasting clones were screened based on the criteria of a |log2 ratio| ≥ 1 and FDR (false discovery rate) ≤ 0.001 by comparing the transcriptome data of the two contrasting cultivars as An vs Bn (n = 0, 1, 2) to identify DGEs related to the different cold-resistance phenotypes of these two rubber cultivars. The data revealed that 2929, 7567 and 5842 genes, respectively, were down-regulated at 0 h, 1 h and 24 h in ‘93–114’ compared with ‘Reken501’, while 1919, 1501 and 1284 genes were up-regulated. In total, fewer genes were expressed in the cold-tolerant ‘93–114’ under control and treated conditions, and more genes were down-regulated during cold stress in ‘93–114’ compared with the cold-susceptible ‘Reken 501.’

The differential gene expression profiles in terms of digital transcriptome analysis were further verified by quantitative real-time PCR. Fourteen randomly selected genes with differential expression between ‘Reken501’ and ‘93–114’ were further selected for qRT-PCR (Real-time quantitative reverse transcription PCR) analysis with specific primers (Supplementary Table S1). The comparison analysis revealed a similar expression tendency under cold conditions for most genes in the experimental qRT-PCR results compared with the digital transcriptome analysis between ‘Reken501’ and ‘93–114’ at 0 h (control condition), 1 h or 24 h (Supplementary Fig. S1).

**Figure 1:**
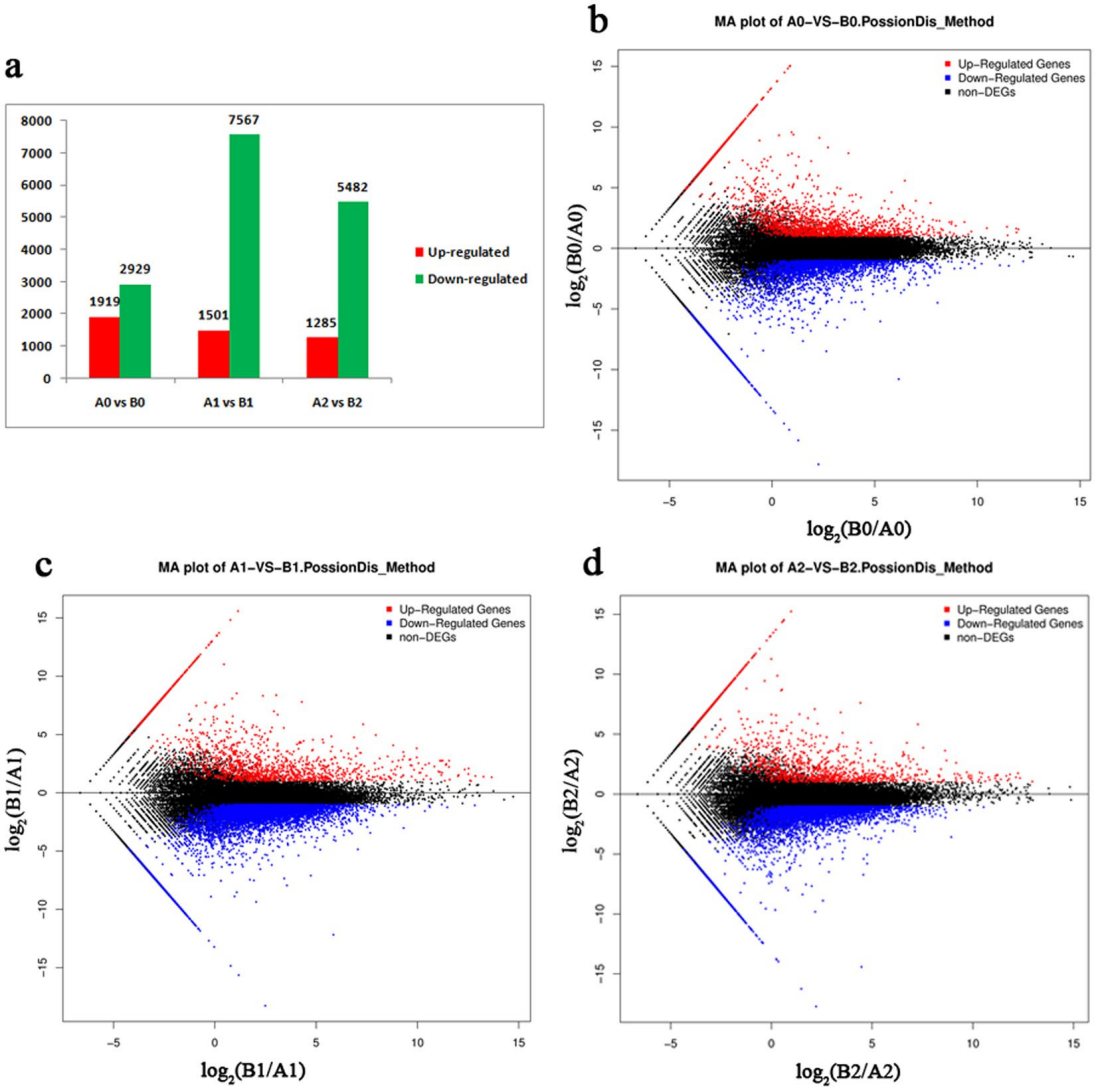
Statistical analysis of differentially expressed genes. (**a**) Numbers of differentially expressed genes between ‘93–114’ and ‘Reken501’ before cold treatment (0 h, A0 vs B0) and after cold treatment for 1 h (A1 vs B1) and 24 h (A2 vs B2). The number of up-regulated (red) and down-regulated (green) genes is shown between ‘93–114’ (B0 for control, B1 for 1 h cold treatment, B2 for 24 h cold treatment) and ‘Reken501’ (A0 for control, A1 for 1 h cold treatment, A2 for 24 h cold treatment). (**b**,**c**) Distribution of differentially expressed genes (FDR ≤ 0.001 and |log2 ratio| ≥ 1).

**Figure 2:**
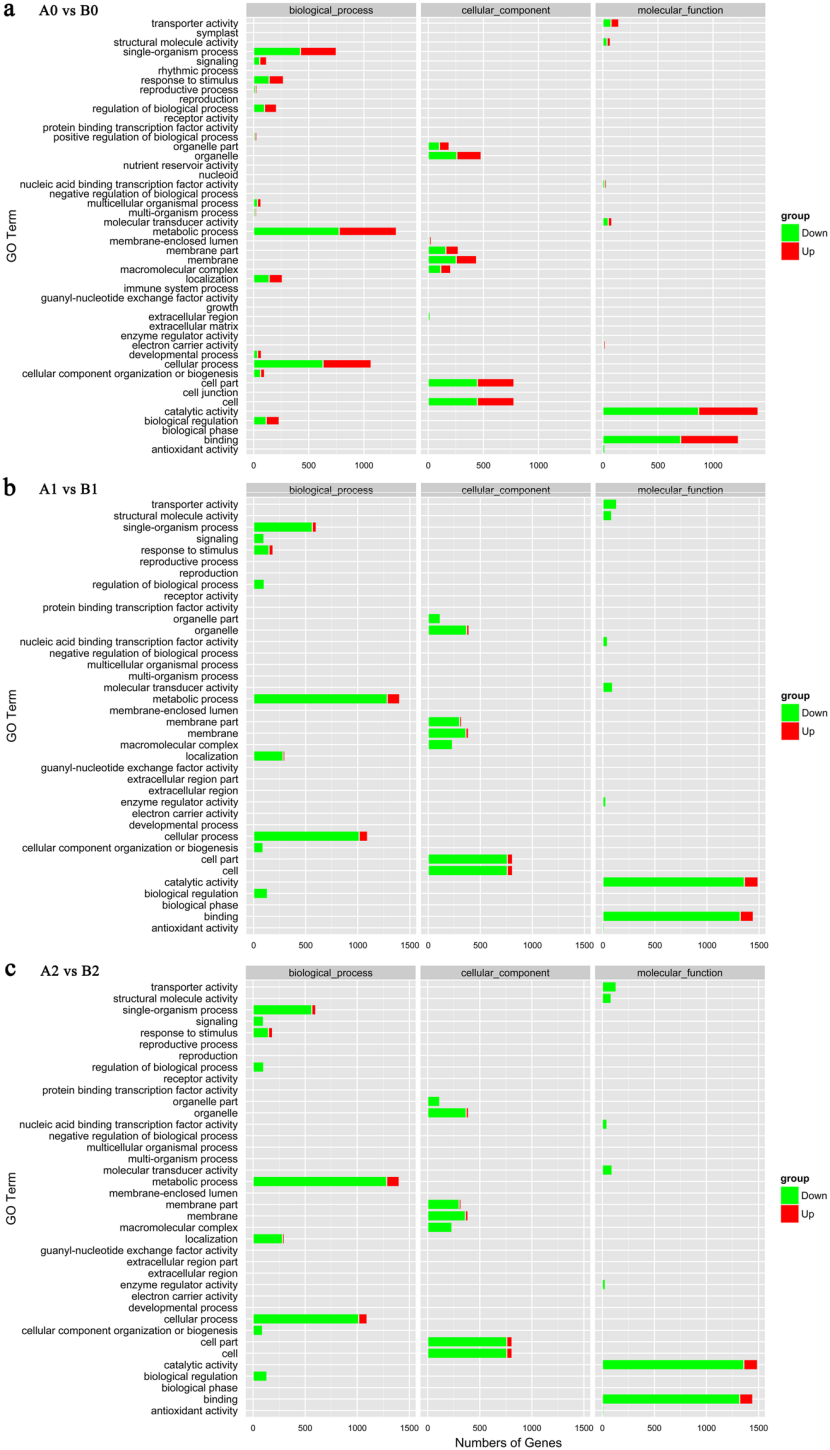
Functional GO annotation of DGEs compared between ‘93–114’ and ‘Reken501’. GO classification analysis of the differentially expressed genes after 0 h (a, A0 vs B0), 1 h (b, A1 vs B1) and 24 h (c, A2 vs B2) of cold treatment. GO categories of ‘biological process,’ ‘cellular component’ and ‘molecular function’ are shown. The up-regulated and down-regulated genes are represented by red and green colors, respectively.

The DEGs were further divided into up-regulated and down-regulated genes and subsequently annotated using the Blast2GO and blast in KEGG (Kyoto Encyclopedia of Genes and Genomes) databases. The DEGs were mainly enriched in ‘binding’ and ‘catalytic activity’ subcategories of the ‘molecular function’ category, in the ‘cellular process’ and ‘metabolic process’ subcategories of the ‘biological process’ category and in ‘cell’ and ‘cell part’ subcategories of the ‘cellular component’ category after 0 h, 1 h and 24 h of cold stress (Fig. 1). During cold stress, despite the repressed expression of most genes in ‘93–114,’ a relatively higher ratio of genes involved in the function of ‘response to stimulus,’ membrane and membrane part, and macromolecular complex retained up-regulated expression in ‘93–114’ compared with ‘Reken 501’ at 1 h and 24 h (Fig. 2). The DEGs were further subjected to GO (Gene Ontology) functional enrichment with a hypergeometric test (Bonferroni correction *P* ≤ 0.05). The anion binding, nucleoside binding, and ribonucleoside binding were enriched GO terms in the category of molecular function after 0 h, 1 h and 24 h of cold stress. The GO term phosphorylation including protein phosphorylation was enriched in the category of biological process in the control and at early stages after 1 h of cold stress. The GO terms regulation of primary metabolic process and cellular macromolecule metabolic process were enriched in the category of biological process after 1 h of cold stress, while the GO terms regulation of macromolecule metabolic process, regulation of transcription, DNA-templated and regulation of biosynthetic process were clearly enriched in the category of biological process at late stages of 24 h of cold stress (Supplementary Table S2).

**Figure 3:**
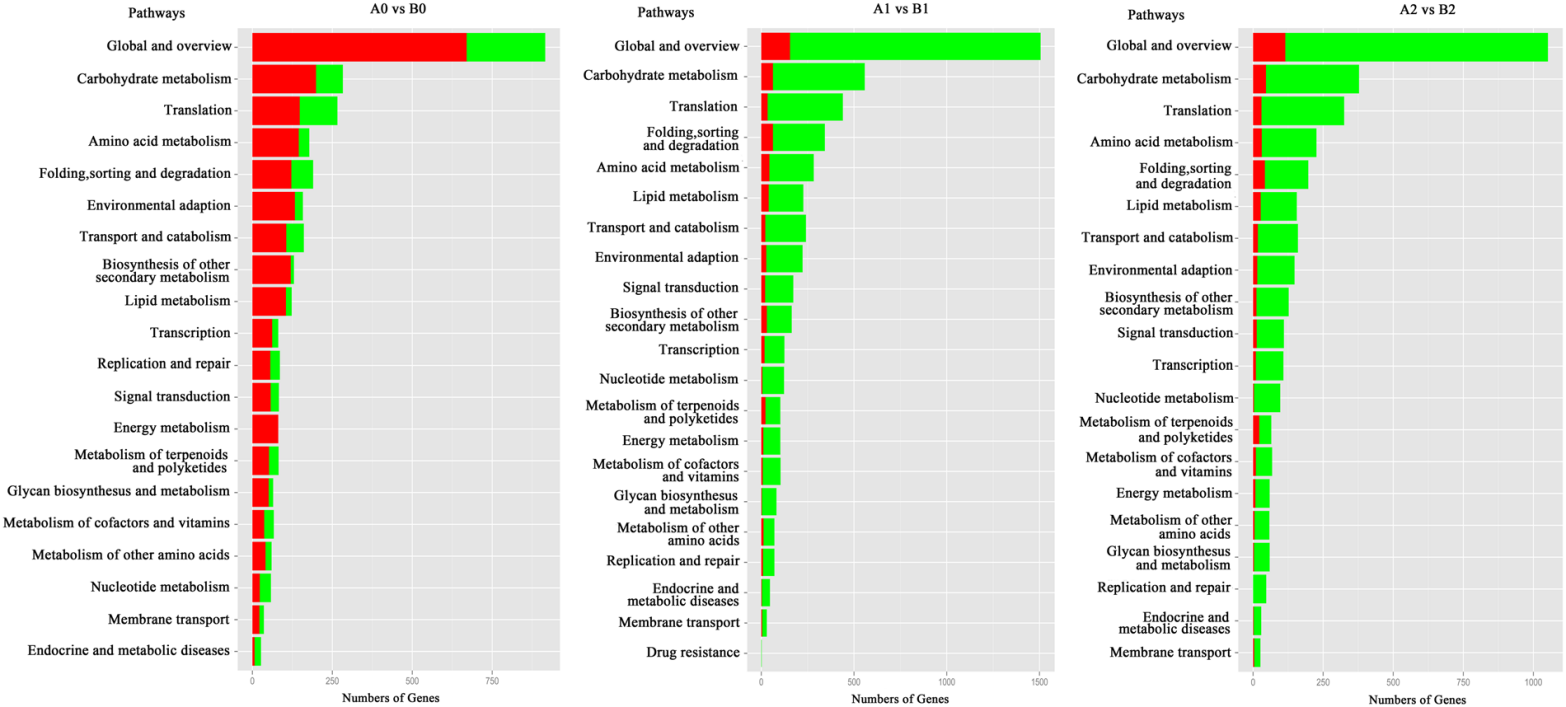
Functional KEGG classification of the DGEs in the pathway analysis between **‘**93-114’ and ‘Reken501’. The functional KEGG classification and enrichment assays of the differentially expressed genes with a hypergeometric test (Bonferroni correction *P* ≤ 0.05) after 0 h, 1 h and 24 h of cold treatment.

When investigating the DEGs in the KEGG pathway, compared with the 0 h control condition, a relatively higher ratio of genes involved in carbohydrate metabolism, folding, sorting and degradation, amino acid metabolism and lipid metabolism retained up-regulated expression in ‘93–114’ in the early and late cold stress stages (Fig. 3). The DEGs were also further subjected to the KEGG pathway enrichment assay with a hypergeometric test (Bonferroni correction *P* ≤ 0.05). In both control and cold stress conditions, the ribosome, peroxisome and proteasome were all enriched to different extents. In the early stage of 1 h of cold stress, protein processing in the endoplasmic reticulum was another enriched KEGG pathway in response to sudden cold stress, while the spliceosome was enriched in response to persistent cold stress for 24 h. Additionally, the biosynthesis of amino acids was also similarly enriched to the 0 h control condition with unknown roles and mechanisms (Supplementary Fig. S2).

### Identification of DEGs involved in JA signaling

JA signaling positively regulates plant responses to cold stress by regulating the activity of the key cold stress regulator ICE1^14,15,36^. Thus, the COI1-JAZ-MYC/bHLH/ICE1-DREB/CBF/EREBP module has been identified as the main JA signal transduction cascade in response to cold stress.

**Figure 4:**
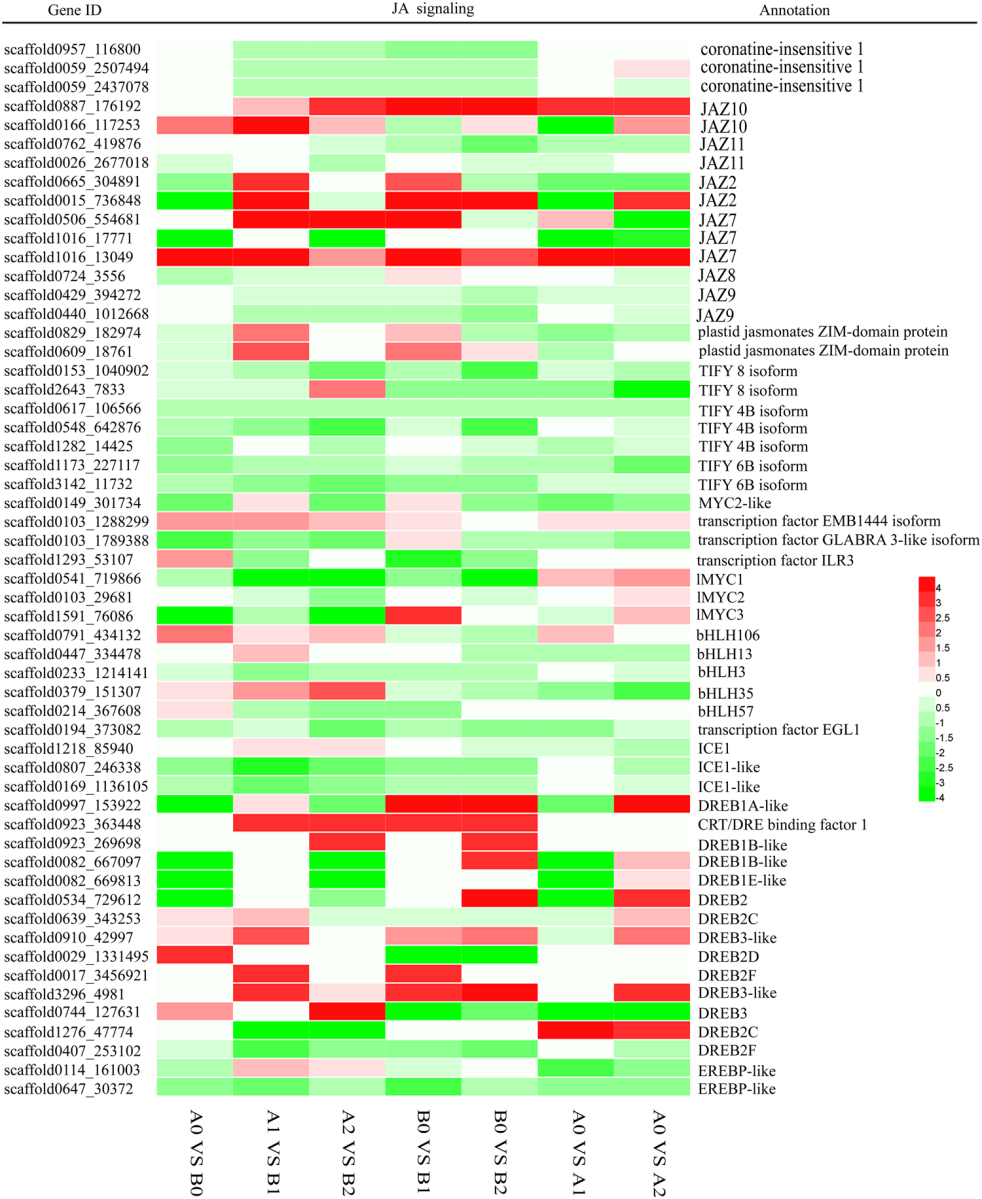
Heatmap of the genes associated with the JA signaling pathway between ‘93–114’ and ‘Reken501’.

As shown in Fig. 4, before cold stress, two JAZ genes (scaffold0166_117253 and scaffold1016_13049), two MYC-type genes (scaffold0103_1288299 and scaffold1293_53107), three bHLH-type (basic helix-loop-helix) genes (scaffold0791_434132 and scaffold0379_151307 and scaffold0214_367608), *DREB2C*, *DREB2D*, *DREB3*, *DREB3-*like and *DREB2F* genes had higher expression levels in ‘93–114’ than in ‘Reken501’. Moreover, after 1 h of cold stress, the expression levels of many genes were altered in response to the cold stress. The expression of the *COI1* genes was lower in ‘93–114’ than in ‘Reken501’ (A1 vs B1) because these genes were inhibited by cold stress in ‘93–114’ (B0 vs B1) but remained unchanged in ‘Reken501’ (A0 vs A1). The *JAZ10* (scaffold0166_117253) and other JAZs (scaffold0665_304891, scaffold0015_736848, scaffold0829_182974 and scaffold0609_18761), MYC2-like, *bHLH13*, *bHLH35*, *ICE1* and several DREB genes (scaffold0923_269698, scaffold0744_127631, scaffold0017_3456921 and scaffold3296_4981), among others, were expressed at higher levels in ‘93–114’ than in ‘Reken501’ (A1 vs B1) because these genes were induced in ‘93–114’ but inhibited or remained unchanged in ‘Reken501’ in response to cold stress. The expression levels of *HblMYC1* and *HblMYC2*, two ICE-like genes (scaffold0807_246338 and scaffold0169_1136105) and *DREB2F* (scaffold0407_253102), were lower in ‘93–114’ than in ‘Reken501’ (A1 vs B1) because the expression levels of these two genes were higher in ‘Reken501’ than in ‘93–114,’ because of their inhibited expression in ‘93–114’ but induced or unchanged expression in ‘Reken 501’ in response to cold stress (B0 vs B1, A0 vs A1). After 24 h of cold stress, many genes were expressed at lower levels in ‘93–114’ compared with ‘Reken501’ because they were inhibited in ‘93–114’ but enhanced in ‘Reken501’, such as COI1 gene (scaffold0059_2507494), *HblMYC1* and *HblMYC2*, *DREB2C* (scaffold0639_343253 and scaffold1276_47774) in response to cold stress, or they were both induced but the expression levels were higher in ‘Reken501’, as observed for JAZ2 (scaffold0015_736848), DREB1A-like (scaffold0997_153922), DREB1B-like (scaffold0082_669813), *DREB2* (scaffold0534_729612). In contrast, DREB1B-like (scaffold_0923_269698) and scaffold0923_363448, and *bHLH106* were expressed at higher levels in ‘93–114’ than in ‘Reken501’ (A2 vs B2) because they were induced in ‘93–114’ but inhibited or remained unchanged in ‘Reken501’. The higher expression of *JAZ7* (scaffold1016_13049), JAZ10 (scaffold0887_176192 and scaffold0166_117253) and DREB 3-like (scaffold3296_4981) genes was due to its higher induction in ‘93–114.’ These results revealed that JA signaling indeed mediated the rubber tree response to sudden cold stress and that several important JAZ, ICE1/bHLH and DREB homologues had selected preferential adaptation roles to sudden cold stress in the cold-tolerant rubber tree clone ‘93–114’ in the non-traditional Hainan province of China where the tress frequently suffer from cold damage.

### Response of heat shock module genes

**Figure 5:**
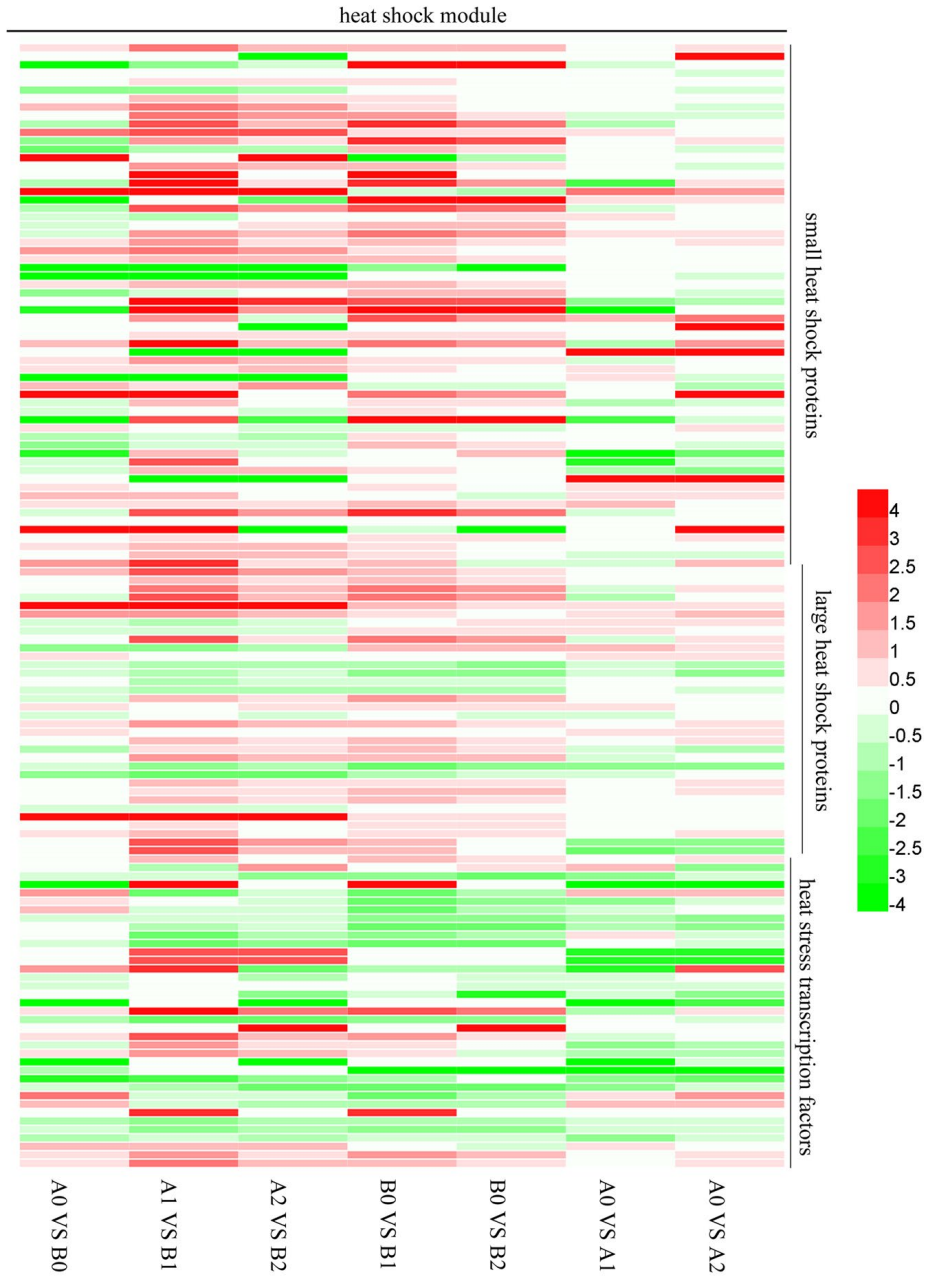
Heatmap of the genes associated with the heat shock module between ‘93–114’ and ‘Reken501’.

Despite the first discovery of the roles in heat stress, the HSP/chaperone network is a major component of multiple stress responses^22^. Increasing data have confirmed that HSPs have the ability to enhance plant tolerance to adverse environmental stresses^37–39^. The HSP family can be divided into HSP15, HSP17–18, HSP21–23, HSP26, HSP70, HSP81–83 and HSP90. Before cold stress, several small and larger heat shock protein genes and most heat stress transcriptional factors were expressed at higher levels in ‘93–114’ compared with ‘Reken501’, while most small and large heat shock proteins genes were expressed at high levels in ‘Reken501’. However, under cold stress, especially at the early stage of 1 h of cold stress, the small heat-shock protein (sHSP) gene, e.g., most of rubber tree *HSP15*, *HSP17.3*, *HSP17.4*, *HSP17.6*, *HSP17.9*, *HSP18.1*, *HSP18.2*, *HSP20*, *HSP21.7*, *HSP22.0* and *HSP23.6* genes, was expressed at higher levels in the cold-tolerant clone ‘93–114.’ The large molecular chaperones *HSP70* and *HSP83* were also enriched in the cold-tolerant clone ‘93–114’ (Fig. 5). These genes were expressed at higher levels in ‘93–114’ due to their induction by cold stress in ‘93–114’ but were inhibited in ‘Reken501’ or exhibited much lower induction levels. Moreover, a few genes had higher expression levels in ‘Reken501’ than in ‘93–114’ because they were induced in ‘Reken501’ by cold stress but down-regulated or induced at lower levels under cold stress conditions (Fig. 5). After 24 h of cold stress, the number of genes with higher expression level was reduced, but the remaining genes were also expressed at relatively higher levels in ‘93–114’ than in ‘Reken501’ with a similar trend in expression. The ‘93–114’ was enriched with small and large HSP/chaperones to protect the stability and activities of its client proteins under sudden cold stress. The protective functions of several important HSP component of rubber tree have been confirmed *in vitro*^40^. HSPs/chaperones are controlled by the action of diverse heat shock factors, making up the Hsf-HSP signaling pathway, in response to multiple stresses. Consistent with the higher expression of HSPs/chaperones in ‘93–114,’ *HsfA1b* (scaffold0154_895105 and scaffold0985_240417), *HsfA4a* (scaffold0262_1081430 and scaffold0476_869883), *HsfA7a* (scaffold0026_252701), *HsfB2a* (scaffold1593_4131), *HsfB2b* (scaffold0118_132983 and scaffold0040_3084829), *HsfB3* (scaffold0887_230318), other types of Hsfs genes (scaffold1045_143160, scaffold1415_90925, scaffold0602_2358 and scaffold0043_1764659) increased to a greater extent in ‘93–114’ after 1 h or 24 h of cold stress (Fig. 5), indicating that these Hsfs might be recruited in response to a low temperature stimulus to provoke the transcription of HSPs/chaperones to eliminate harmful molecules and renew order in whole cells.

### Response of ROS scavenger genes

**Figure 6:**
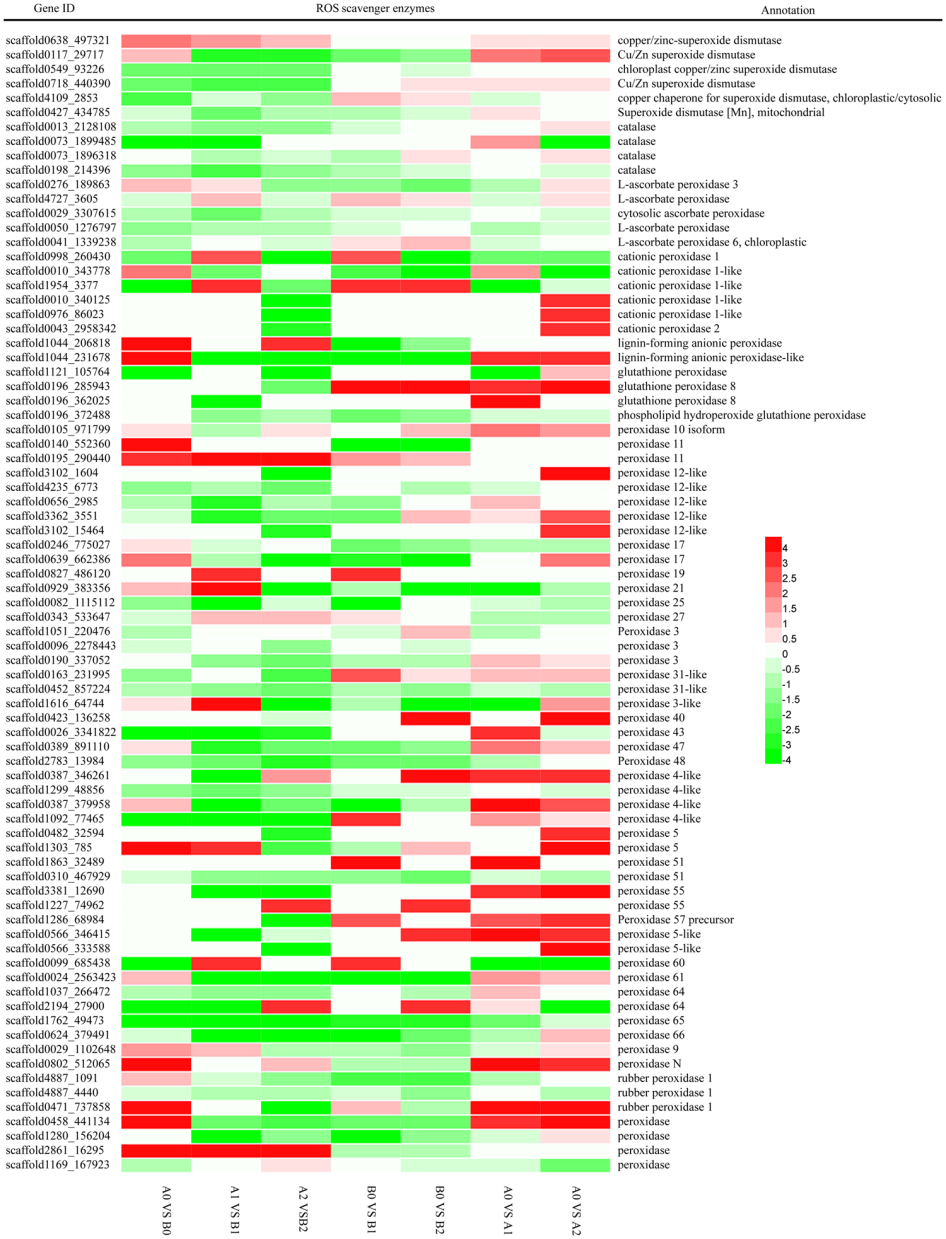
Heatmap of the ROS scavenging enzyme genes between ‘93–114’ and ‘Reken501’.

ROS are generated during abiotic stresses in plants, resulting in a redox imbalance and oxidative stress. ROS scavengers, especially the major detoxification enzymes SOD (superoxide dismutase), POD (peroxidase) and CAT (catalase) are activated in response to environmental stresses^33,41,42^. Generally, most SOD-encoding genes and all the identified CAT-encoding genes showed lower expression levels in ‘93–114’ than in ‘Reken501’, except for one scaffold0638_497321 encoding SOD, which showed significantly higher expression in ‘93–114’ after 0 h, 1 h and 24 h of cold treatment. The transcript level of this SOD gene (scaffold0638_497321) was still higher in ‘93–114,’ although it was slightly down-regulated in response to cold stress for 1 h and 24 h in ‘93–114’ while being induced in ‘Reken501’ (Fig. 6). Additionally, the number of POD-encoding genes showed differential expression to a greater extent than the above SOD- and CAT-encoding genes despite the detailed up- or down-regulated expression trends. In detail, several different types of peroxides exhibited a sharp change in expression before or during early or late cold stress stages between these two contrasting cold-tolerant rubber clones. Under normal growth condition, the cationic peroxidase 1-like (scaffold0010_343778), lignin-forming anionic peroxidase (scaffold1044_206818), lignin-forming anionic peroxidase-like (scaffold1044_231678), peroxidase 11 (scaffold0140_552360), peroxidase 5 (scaffold1303_785), peroxidase N (scaffold0802_512065) and peroxidase (scaffold2861_16295) genes had significantly higher basal expression levels in ‘93–114.’ At the early cold stress stage, thirteen POD-encoding genes were expressed at relatively higher levels, seven of which also showed an induction of expression in ‘93–114’ but a down-regulation or no significant change in expression in ‘Reken501’ after 1 h of cold stress. The remaining POD genes were down-regulated or showed no change in expression in ‘93–114,’ but most of them showed induced expression in ‘Reken501’ after 1 h of cold stress. For example, L-ascorbate peroxidase (scaffold4727_3605), cationic peroxidase 1 (scaffold0998_260430 and scaffold1954_3377), peroxidase 11 (scaffold0195_290440), peroxidase 19 (scaffold0827_486120), peroxidase 21 (scaffold0929_383356), peroxidase3-like (scaffold1616_64744), peroxidase 60 (scaffold0099_685438), peroxidase 9 (scaffold0029_1102648), and peroxidase (scaffold2861_16295) showed significantly higher expression in cold-tolerant ‘93–114’ at the 1 h early cold stress stage. The expression of peroxidase 19 was induced in ‘93–114’ but was slightly down-regulated in ‘Reken501’ after 1 h of cold stress; peroxidase3-like and peroxidase 60 genes showed trends toward induced expression in ‘93–114’ but slightly down-regulated trends in ‘Reken501’ after 1 h of cold stress. Similarly, lignin-forming anionic peroxidase (scaffold1044_206818), peroxidase 11 (scaffold0195_290440), peroxidase 27 (scaffold0343_533647), peroxidase 4-like (scaffold0387_346261), peroxidase55 (scaffold1227_74962), peroxidase 64 (scaffold2194_27900), and peroxidase (scaffold2861_16295) had higher expression levels in cold-tolerant ‘93–114’ at the 24 h late stage cold response (Fig. 6). Peroxidase 27 and peroxidase55 were induced by cold stress in ‘93–114’ but inhibited in ‘Reken501’. The peroxidase 4-like gene was induced in both ‘93–114’ and ‘Reken501’ by cold stress but showed relatively higher expression in ‘93–114’ than in ‘Reken501’. Collectively, these data indicated that the POD enzymes might play significant roles in eliminating toxic ROS to protect cells from oxidative damage, and their functions may be stronger in cold-tolerant ‘93–114.’

### Identification of DEGs involved in ethylene signaling

ET signaling pathway has been shown to have a negative role in regulating freezing tolerance in *Arabidopsis* by repressing the CBF pathway^16,43^. Under normal growth condition, the ethylene receptor (scaffold0979_291486), *EIN4* (Ethylene insensitive) (scaffold0239_551511), *CTR1* (Constitutive triple response1) (scaffold1663_22838), EIN2-type genes (scaffold0007_1670663), *EBF* (scaffold0690_138377), as well as *TIFY* gene (scaffold0754_396540) and an ethylene-insensitive protein gene (BGI_novel_G001186) had higher expression levels in ‘93–114’ (A0 vs B0) (Supplementary Fig. S3). After 1 h of cold stress, apart from three EIN2 genes had higher expression levels in ‘93–114’; other genes involved in ethylene signaling had lower expression levels in ‘93–114’ than in ‘Reken501’ because most of these genes were down-regulated in ‘93–114’ but up-regulated or remained unchanged in ‘Reken501’ in response to cold stress, or the levels of expression were unchanged in ‘93–114’ but inhibited in ‘Reken501’ by cold stress. Similarly, after 24 h of cold stress, the ethylene receptors, most *CTR1* genes, four *EIN2* (scaffold0093_375297, scaffold0050_2714677, BGI_novel_G002819 and BGI_novel_G002818), *EIN3* and *EBF* (EIN3-binding F-box protein) genes, *NTF4* gene and mc410 gene had lower expression levels in ‘93–114’ than in ‘Reken501’ because they were inhibited both in ‘93–114’ and ‘Reken501’ after cold stress. Moreover, two *EIN2* genes (BGI_novel_G002818 and BGI_novel_G002819) and two *TINY* genes (scaffold0864_20762 and scaffold0754_396540) also displayed lower expression levels in ‘93–114’ than in ‘Reken501’ because the induction level in ‘93–114’ in response to cold stress was much lower than that in ‘Reken501’. These results indicated that the cold-tolerant ‘93–114’ clone repressed the ethylene signaling response during cold stress.

### Auxin and ABA signaling in responses to low temperature stress

Auxins not only have vital roles in regulating plant development, but they also mediate abiotic stress responses^44^. The TIR1 (Transport inhibitor response 1) -Aux/IAA (Indoleacetic acid-induced protein) -ARF (Auxin response factor) module consists of auxin signal transduction in response to *in vivo* and *in vitro* signals^45^. During normal conditions without cold stress, the auxin transporter AUX1(Auxin transporter protein), auxin receptor TIR1, repressors Aux/IAAs and positive activators ARFs, as well as the early auxin response gene GH3 (Gretchen hagen), showed differential expression patterns. Among these, the higher expression of the repressors Aux/IAAs in ‘93–114’ may indicate a lower rate of growth and development compared with another rubber tree clone, ‘Reken501’. During early exposure to low temperature stress, despite the lower expression of *TIR1* and *ARFs*, the repressors Aux/IAAs and activators ARFs were clearly down-regulated in ‘93–114’ at the early 1 h cold stress stage. Most of these genes with lower expression levels in ‘93–114’ were down-regulated in response to cold stress, but they were oppositely up-regulated or unchanged by cold stress in ‘Reken501’. *ARF1* gene (scaffold0375_663805), two AUX/IAA28 genes (scaffold0319_1118315 and scaffold0168_1797736), and one *SAUR* gene (scaffold0932_96711) were slightly higher in ‘93–114’ than in ‘Reken501’ at the 1 h early stress stage (Supplementary Fig. S4). At the late 24 h cold stage, genes encoding the repressors *AUX/IAA28* (scaffold0168_1797736), *AUX/IAA29* and *TIR1* (scaffold0492_67897) exhibited relatively higher expression in ‘93–114’ than in ‘Reken501’, in which TIR1-encoding genes was down-regulated in ‘93–114’ and up-regulated in ‘Reken501’ in response to cold stress, but the expression levels of *AUX/IAA28* (scaffold0168_1797736) and AUX/IAA29 genes were also higher in ‘93–114’ than in ‘Reken501’ although there were inhibited in both of the rubber clones by cold stress, as revealed by the RSEM (RNA-Seq by expectation maximization) value. Apart from these genes, the remaining genes shown in Supplementary Fig. S4 had lower expression in ‘93–114’ than in ‘Reken501’, displaying a down-regulated trend in expression in ‘93–114’ and a up-regulated trend (AUX/IAA4, *AUX/IAA28* (scaffold0319_1118315), *AUX/IAA14*, *AUX/IAA26*, *AUX/IAA27*, *ARF4* and *ARF8*) or a down-regulated trend with a higher RSEM value or a unchanged trend in ‘Reken501’ after 24 h of cold stress.

Similarly, under normal conditions, most ABA signaling genes had relatively lower expression levels or unchanged in expression in ‘93–114’. After 1 h of cold stress, excluding the ABA receptor PYL4 (Pyrabactin resistence) and *bZIP53* (basic leucine zipper), most ABA signaling genes, including the ABA receptor *PYL9*, PP2C-type (protein phosphatases type 2C) genes and the SnRK2 (Sucrose non-fermenting 1-related protein kinase 2) kinases and activators ARFs, were expressed at higher levels in ‘Reken501’ than in ‘93–114.’ PYL4 was up-regulated by cold stress in ‘93–114’ but its expression level was unchanged in ‘Reken501’. The remaining genes shown in Supplementary Fig. S5 with lower expression levels in ‘93–114’ after 1 h of cold stress were down-regulated by cold stress in ‘93–114’ (B0 vs B1) but up-regulated or remained unchanged in ‘Reken501’ (A0 vs A1). Similarly, most of the genes encoding PYL9, repressors PP2Cs and activators SnRK2 kinases and transcriptional factor ARFs were expressed at lower levels in ‘93–114’ than in ‘Reken501’ at the late 24 h cold stress stage (A2 vs B2). Most of these genes were inhibited by cold stress in ‘93–114’ (B0 vs B2) but induced or remained unchanged in ‘Reken501’ (A0 vs A2), as shown in Supplementary Fig. S5. This ABA signaling expression pattern might suggest that the cold-tolerant clone ‘93–114’ did not have preferentially strengthened ABA signaling in response to cold stress. In contrast, the cold-susceptible clone ‘Reken501’ showed activated ABA signaling to cope with the sudden low temperature stress.

### Metabolic changes and protein degradation in responses to cold stress

When encountering environmental stresses, plants proactively rearrange their metabolic activities to adapt to the altered conditions. In this case, the rubber cold-tolerant clone ‘93–114’ sustained a relatively higher transcription level of genes involved in flavonoid biosynthesis than ‘Reken501’ after 1 h and 24 h of cold stress, similar to the control condition. In greater detail, genes involved in the biosynthesis of dihydrokaempferol, dihydroquercetin, and dihydromyricetin, as well as their products epiafzelechin, epicatechin, and epigallocatechin, were significantly increased in the rubber cold-tolerant clone ‘93–114’ than in ‘Reken501’ under control and cold stress conditions. These genes encode flavonoid 3′, 5′-hydroxylase [EC:1.14.13.88] (scaffold0251_1443705, 4.8 times), flavonoid 3′-monooxygenase [EC:1.14.13.21] (scaffold0746_184319, 2.7 times), bifunctional dihydroflavonol 4-reductase/flavanone 4-reductase [EC:1.1.1.219/1.1.1.234] (scaffold0823_94491, 2.7 times; scaffold0823_72771, 1.7 times), leucoanthocyanidin dioxygenase [EC:1.14.11.19] (scaffold0912_372950, 8.3 times; scaffold3044_17076, 6.7 times), scaffold4462_1282, 3.9 times), and anthocyanidin reductase [EC:1.3.1.77] (scaffold0142_646036, 1.8 times) (Supplementary Fig. S6). Among these, flavonoid 3′, 5′-hydroxylase, flavonoid 3’-monooxygenase and anthocyanidin reductase were up-regulated in ‘93–114’ but down-regulated or remained unchanged after 1 h or 24 h of cold stress. The expression of the leucoanthocyanidin dioxygenase-encoding gene (scaffold0912_372950 and scaffold4462_1282) was enhanced by cold stress in both ‘93–114’ and ‘Reken501’, but at relatively higher expression levels in ‘93–114’ than in ‘Reken501’. Dihydrokaempferol, dihydroquercetin, dihydromyricetin, epiafzelechin, epicatechin, and epigallocatechin are vital antioxidants during ROS dysfunction. Moreover, epiafzelechin, epicatechin, and epigallocatechin can further form proanthocyanidins, which are some of the most efficient antioxidants in plants. Together with the POD enzymes, these antioxidants constitute effective ROS scavengers in response to harmful cold stress effects. By contrast, many genes involved in pyrimidine and purine metabolism were significantly lower in ‘93–114’ than in ‘Reken501’ after 1 h and 24 h of cold stress, as shown in the supplemental figures (Supplementary Figs S7, S8). Similar lower expression levels were observed for genes encoding subunits of the ubiquitin ligase complex and proteasome, which participate in the ubiquitin-mediated protein degradation process (Supplementary Figs S9, S10).

### Expression of stress-related genes in response to chilling stress

**Figure 7:**
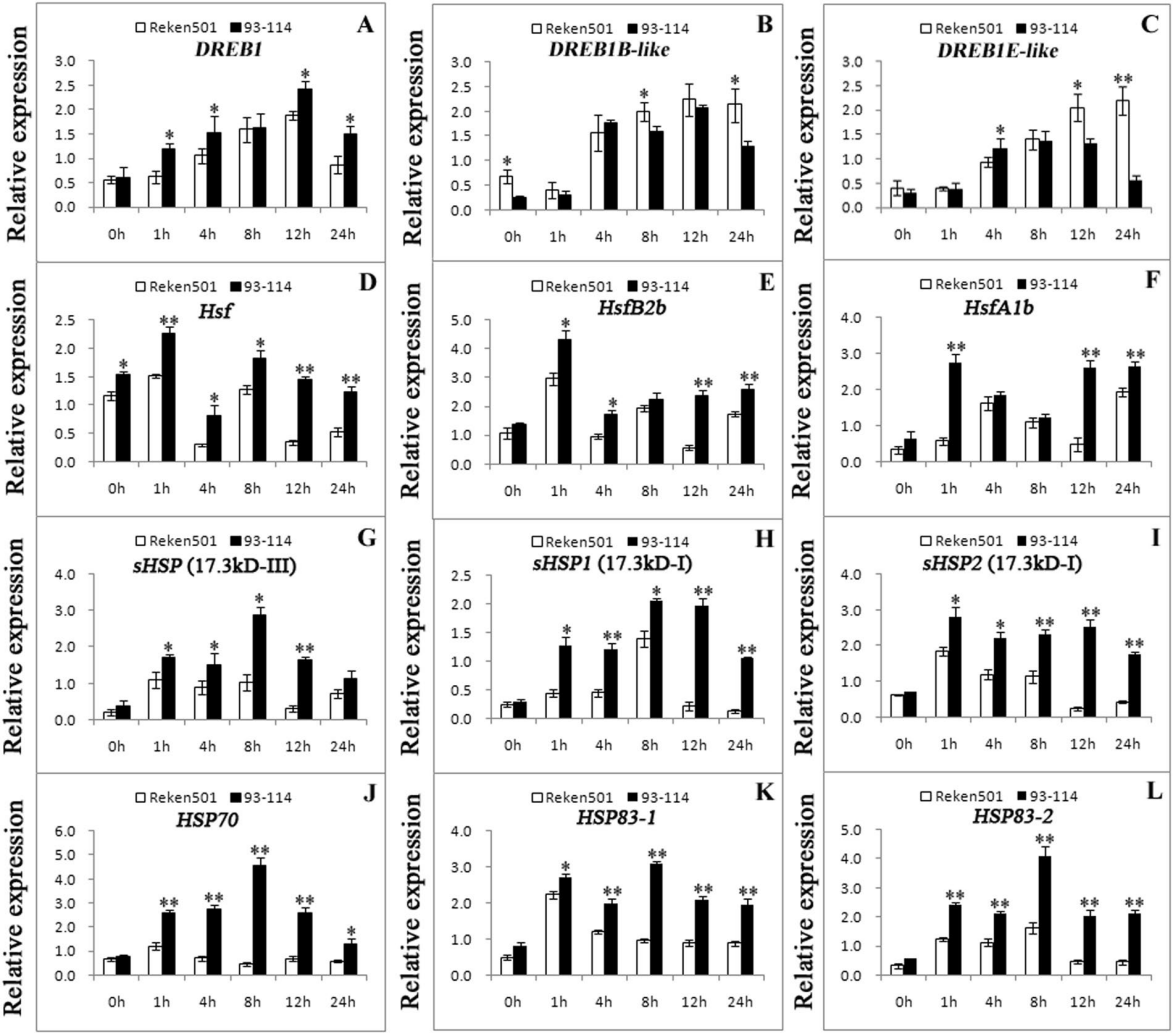
The qRT-PCR analysis of stress-related genes in the upstream analysis at different time intervals before and after cold treatment. The relative expression of each gene was calculated as the 2^−ΔΔCT^ value and normalized to the endogenous reference genes. The standard deviation of three biological replicates is indicated by 1 or 2 asterisks depending on the *P* value for the significant difference (*P* < 0.05, *P* < 0.01, respectively) after *t*-test analysis (two group comparisons).

To further investigate the expression profiles of the identified stress-related genes in the upstream analysis, qRT-PCR was used to detect stress-related genes at different time intervals before and after cold treatments. As shown in Fig. 7 and Supplementary Fig. S11, the *DREB1* and *DREB3* genes showed higher expression in ‘93–114’ than in ‘Reken501’ during cold stress, while *DREB1B-like*, *DREB1E-like*, *DREB2C* and *DREB2F* genes had higher expression in ‘Reken501’. The MYC2-like gene, a bHLH-type transcriptional factor in JA signaling, was also induced by chilling stress and was expressed at higher levels in cold-tolerant ‘93–114,’ suggesting its important roles in regulating downstream target stress genes that respond to chilling stress. Moreover, the expression levels of the ethylene singling pathway genes *ETR2* (Ethylene response), *CTR1* and *EIN3* were higher in cold-susceptible ‘Reken501’ during chilling stress, supporting their negative roles in chilling stress responses. Several Hsf-HSP module genes, such as *Hsf*, *Hsf1b* and *HsfB2b* as well as the sHSP17.3 type protein genes, *HSP70*, *HSP83–1* and *HSP83–2*, were cold inducible and had relative higher expression levels in cold-tolerant ‘93–114’ than in cold-susceptible ‘Reken501’ during chilling stress. These results suggested that these genes were more activated in cold-tolerant ‘93–114’ during chilling stress and fulfilled their protective function of macromolecules in cells to attenuate or relieve the harmful damage to leaf cells caused by chilling stress. Interestingly, the expression profiles of cold-inducible genes with higher expression in cold-tolerant ‘93–114’ than in cold-susceptive ‘Reken501’ included the mitogen-activated protein kinase kinase *MKK6* (mitogen-activated protein kinase kinase 6) gene, indicating that MAPKKK (mitogen-activated protein kinase kinase kinase) -MAPKK-MAPK (mitogen-activated protein kinase) cascades might also play a vital role in the regulation of chilling stress tolerance, as confirmed by previous reports showing that *OsMKK6DD* and *ZmKK1* over-expression enhances cold stress tolerance in plants^46,47^. Additionally, the MAPKK-MAPK cascade can activate Hsf transcription factors to control downstream HSP expression during stress responses, which connect MAPKKK-MAPKK-MAPK signaling cascades with the Hsf-HSP regulons^32^.

## Discussion

Deep sequencing of approximately 5–8 Gb of reads ensures approximately 90% transcriptome coverage for the rubber tree with complex genetics^48^. In the present study, approximately 7.4 Gb clean reads were obtained from each of the six distinct sequencing leaf samples of the two contrasting cold-tolerant rubber tree clones, equivalent to a previously described sequencing depth (7–8 Gb)^49^. Thus, the present sequencing depth contains high-quality bases for further investigation. Due to the availability of published fine rubber tree genome sequences, we mapped all the clean reads onto the rubber tree genome reported by Tang *et al*. (2016)^1^, and approximately 89.87% and 72% reads, respectively, were mapped onto the rubber tree genome and the rubber tree gene sets for each sample, suggesting that the samples were comparable. To our knowledge, this is the first attempt to analyze rubber tree transcriptome data using the referenced rubber tree genome sequences. Therefore, the identification of differentially expressed genes by using the referenced genome is beneficial for more precisely calculating the expression of annotated genes and eliminating the ambiguous identification of homologous genes in the two rubber tree cultivars ‘93–114’ and ‘Reken501’.

In this study, as the cold-tolerant and cold-susceptible phenotypes of the rubber tree cultivars ‘93–114’ and ‘Reken501’ were confirmed, we extracted DGEs between ‘93–114’ and ‘Reken501’ after 0 h, 1 h (early cold stress stage) and 24 h (late cold stress stage) to specifically identify real DGEs associated with the differential phenotypes of these two typical rubber tree clones. Using this strategy, we found several plant signaling pathways and stress-responsible modules that showed a significant difference before and upon cold stress. These findings indicated that the genetic variation of ‘93–114’ and ‘Reken501’ in nature and different responses to cold stress were part of the reasons for the different survival of these two contrasting clones during cold stress. For example, when plants are exposed to stress conditions, the growth rate sharply declines, and stress responses are activated to rearrange biological activities for survival^50^. The intracellular auxin gradient is therefore modified to different degrees by auxin homeostasis upon abiotic stresses. Cold stress inhibits plant growth and development by altering the intracellular auxin gradient^50,51^. To our surprise, different cultivars of the rubber tree, a woody plant, showed opposite auxin signaling responses during cold stress. The cold-tolerant rubber tree clone ‘93–114’ showed enhanced auxin responses under normal growth conditions but inhibited auxin responses immediately after cold stress like most plant responses to cold stress. However, the cold-susceptive rubber tree clone ‘Reken501’, the germplasm of which was introduced from Malaysia, seemed to exhibit nonspecific growth regulation during cold stress, as auxin signaling transduction remained activated during cold stress (Supplementary Fig. S4). Therefore, the sharp inhibition in growth and auxin signaling might be a characteristic of the cold-tolerant rubber tree clone ‘93–114.’

Moreover, flavonoids are natural antioxidants in plants^52,53^, and the expression of genes involved in the metabolism of flavonoids, including flavonoid 3′,5′-hydroxylase, flavonoid 3′-monooxygenase, bifunctional dihydroflavonol 4-reductase/flavanone 4-reductase, leucoanthocyanidin dioxygenase and anthocyanidin reductase, were elevated in ‘93–114’ both before and after cold stress, suggesting that enhanced flavonoid biosynthesis was a positive trait of ‘93–114’ for survival during natural selection on farms during exposure to natural cold stress. Our previous study of the differential physiological changes between ‘93–114’ and ‘Reken501’ has revealed lower content of MDA (Malondialdehyde), an indicator of the oxidation of lipids in membrane^54^, in ‘93–114’ during cold stress. The lower content of MDA indicates a lower degree of ROS damage in ‘93–114’ under cold stress. Indeed, the transcript levels of several ROS-scavenging enzymes, POD genes, were significantly higher in ‘93–114’ after 1 h or 24 h of cold stress. These higher transcriptional levels of PODs may be partly regulated by the heat stress shock module during cold stress, as similar patterns of higher expression of Hsf and HSP genes were also observed in ‘93–114.’ Increasing evidence has shown that ROS-scavenging enzymes can be mediated at the transcriptional level by the heat stress shock module. In *Arabidopsis*, overexpression of *AtHSF3* enhances the transcript levels of *Apx*^*S*^ and ascorbate peroxidase encoding the *AtApx2* gene^55^. Ectopic expression of *CtHsfA2b* in *Arabidopsis* enhances the transcriptional activity of *AtApx2* by direct binding to the heat shock element (HSE) on the promoter of *AtApx2*^33^. In contrast, Hsf transcriptional factors can stimulate the expression of ROS scavenging enzymes and HSPs^33,55^; Hsfs can be phosphorylated and activated by the MAPKK-MAPK cascade, and activated Hsfs in turn provoke downstream HSP expression^32^. However, HSPs can in turn retain the stability and activities of the Hsfs and ROS-scavenging enzymes, and they might ultimately form a positive feedback loop in response to external stresses^22,32,45,55,56^. Thus, possible formation of the Hsf-HSPs/POD-regulating module in ‘93–114’ was enhanced in response to cold stress. In plants, *Hsfs* and *HSPs* genes are the targets of the DREB-type transcription factors^26,57^. DREB genes, including some *DREB1* and *DREB3* genes, were expressed at higher levels in ‘93–114,’ suggesting their vital positive roles in regulating downstream targets of functional proteins to protect ‘93–114’ rubber tree clones from cold stress damage. Overexpression of DREB-type genes enhances stress tolerance in transgenic plants^58–62^. In particular, the cold-inducible *HbICE1* and *HbCBF1* genes from the cold-tolerant rubber tree clone ‘93–114’ enhance cold tolerance, respectively, in yeasts and transgenic *Arabidopsis*^21,63^. Similarly, like the tropical rubber trees, cassava is also a tropical plant and belongs to the Euphorbiaceae. Overexpression of *MeCBF1* enhances cold tolerance in cassava^59^, suggesting that the CBF-regulon also functions in tropical plants of Euphorbiaceae. Some DREB/CBF genes play important roles at downstream crosstalk nodes of JA and ABA signaling during plant stress responses^17^. Although JA and ABA are two positive plant stress phytohormones^14,15,36,64,65^, many ABA signaling components encoding genes, e.g., ABA receptors such as PYLs, negative regulator PP2C, positive regulator SnRK2, and downstream transcriptional regulators such as ABF-encoding genes had lower transcript levels in ‘93–114’ compared with ‘Reken501’ under cold stress; however, the important signaling component genes *JAZ1*, *JAZ2*, *JAZ7* and *JAZ10* and *ICE1*, *bHLH35* and *bHLH106* had higher expression levels in ‘93–114’ than in ‘Reken 501’, although they were slightly down-regulated after cold stress in ‘93–114.’ These findings indicated that the cold-tolerant ‘93–114’ preferentially selected enhanced JA signaling but not ABA signaling as the main upstream signaling response to cold stress signals. Interestingly, DREB/CBF genes are repressed by the positive component EIN3 in ethylene signaling during cold stress in *Arabidopsis*^16^, partially explaining the negative effect of regulating freezing tolerance via ethylene signaling. In accordance with this possible negative regulatory mechanism, the negative effect of ethylene signaling may be attenuated in ‘93–114’ compared with ‘Reken501’ by most ethylene signaling genes, e.g., most ethylene receptor genes, CTR1 genes, EIN2 and EIN3 genes had lower expression levels in ‘93–114’ than in ‘Reken501’ after 1 h and 24 h of cold stress. The strategies adopted by the cold-tolerant clone ‘93–114’ included strengthening of the positive effect of JA signaling by enhancing the DREB-Hsf-HSP-POD cascade while attenuating the negative effect of ethylene signaling by relieving the repressed expression of DREBs by EIN3. Collectively, the present genome-wide transcriptome response comparative provides cues for the elucidation of the molecular mechanisms underlying cold tolerance and the identified vital differentially expressed genes may be beneficial for the genetic improvement of *H. brasiliensis* clones.

## Methods

### Sampling and cold treatment

Seedlings of *H. brasiliensis* cultivated variety ‘Reken501’ and ‘93–114’ were grown at the experimental station of the Rubber Research Institute, Chinese Academy of Tropical Agricultural Sciences, P.R. China. Cold treatments were performed when the seedlings developed two extension units and the leaves were completely mature. A total of six batches were assessed, of which two batches without cold treatment (28 °C) were regarded as controls (A0 for ‘Reken501’; B0 for ‘93–114’). Two batches of each rubber tree clone were subjected to 4 °C treatment for 1 h (A1 for ‘Reken501’; B1 for ‘93–114’) and two batches of each rubber tree clone were subjected to 4 °C treatment for 24 h (A2 for ‘Reken501’; B2 for ‘93–114’). The leaf samples were collected for RNA-seq from the six batches of samples. Nine leaves was pooled for each leaf sample from three biological rubber tree clones, in which three leaves were collected from one biological rubber tree clone. For qRT-PCR assay, another different period of experimental material samples were used, and the methods of untreatment and cold treatments were the same as mentioned above, but the sample points were increased into six time courses (0 h, 1 h, 4 h, 8 h, 12 h, and 24 h). The collected leaf samples were frozen immediately in liquid nitrogen and stored at −80 °C for subsequent use.

### RNA extraction and library preparation for transcriptome analysis

Total RNA was extracted from the collected leaves using the CTAB extraction and LiCl precipitation methods according to previous reports with slight modifications^66,67^. Then, the totals RNA were further purified using the TURBO DNA-free™ Kit (Ambion) to completely remove genomic DNA contamination. Total RNA concentration was quantified using a NanoDrop ND-1000 spectrophotometer. The RNA quality was analyzed using an Agilent 2100 Bioanalyzer and further verified on 1.0% denaturing agarose gels. The cDNA library construction for further Solexa sequencing analysis was performed at the Beijing Genomics Institute (BGI; Shenzhen, China). Approximately 20 μg of total RNA from each sample pool of the two clones, ‘Reken501’ (A0, A1 and A2) and ‘93–114’ (B0, B1 and B2), was used for cDNA library construction with standard protocols. The detailed process was described in a previous research study^60^. Briefly, six independent cDNA libraries were constructed and sequenced using an Illumina HiSeq. 2000 genome analyzer.

### Sequencing and mapping of Illumina reads and data analysis

The methods used for data filtering of the present *H. brasiliensis* transcriptome were performed in the BGI and were very similar to those previously described^68^. The raw reads generated by the Illumina HiSeq. 2000 genome analyzer were subsequently analyzed by FastQC (http://www.bioinformatics.babraham.ac.uk/projects/fastqc/) to determine the base quality and cleaned by removing adaptor sequences, low-quality sequences including empty reads, and sequences containing > 10% bases with a Phred quality score < 20. Any potential polluting foreign sequences (such as fungi) were determined by comparison with the nt database. The best aligned sequence (e < 1e^−10^ and coverage > 80%) was chosen for further use. In this case, most sequences were compared with the rubber tree genomes. No evident pollution was found in the six samples. The remaining clean reads were mapped to the reference rubber genome sequence published in 2016 using HISAT^28^. It is noteworthy that the published rubber tree genome sequence was further annotated in BGI. The identified novel genes were designed with the mark as in BGI, which were differentiated from the mark as a scaffold. After genome mapping, StringTie^69^ was used to reconstruct the transcripts, and cuffcompare^70^ was used to identify novel transcripts in the samples with genome annotation information. After detecting the novel transcripts, we merged the novel coding transcripts with the reference transcripts to obtain a complete reference, which served to map the clean reads using Bowtie2^71^, followed by calculation of the gene expression level for each sample with RSEM^72^.

### Functional annotation of differential gene expression

In the present study, two important criteria, |log_2_Fold change ≥ 1| for at least one stage comparison and FDR ≤ 0.001), were used as the thresholds to assess significant differences in gene expression in the two rubber clones among the three stages (before cold treatment, after 1 h cold treatment, and after 24 h cold treatment). All differentially expressed genes between treated and control samples from each clone (A0 vs B0 and A1 vs B1, A2 vs B2 represented for 0 h, 1 h and 24 h treatment), serving as differential gene expression datasets between these two contrasting rubber clones, were further subjected to functional annotation and enrichment analysis in the public nr (non-redundant protein sequences), GO (http://www.geneontology.org)^73,74^ and KEGG databases (http://www.genome.jp/kegg)^75–77^ using the BLASTx (Basic local alignment search tool) program (E-value threshold 10^−5^). Additionally, the identification of DGEs from comparisons of cold-treated and control rubber tree clones ‘93–114’ (B0 vs B1 and B0 vs B2) or ‘Reken501’ (A0 vs A1 and A0 vs A2) were also conducted using the above methods.

### Real-time PCR analysis

RNA extracted from leaves was obtained following the protocols supplied with the TIANGEN RNA isolation kit (TIANGEN, Beijing, China). Next, 1 μg of total RNA from each sample of ‘Reken501’ and ‘93–114’ was used for cDNA synthesis using standard protocols. For real-time PCR analysis, gene-specific primers of selected differentially expressed genes were designed using Primer5 software (Supplementary Table S1). All the gene primers were first tested for specificity by RT-PCR. The RT-PCR products were verified by agarose gel electrophoresis and sent for sequencing before being used for subsequent real-time PCR analysis. qRT-PCR was performed with the CFx384TM Real-Time System (Bio-Rad, USA) using a SYBR Premix Ex Taq TM II kit (Takara, Japan). Several internal control genes were also analyzed, and five internal reference genes (*UBC2a*, *YLS8*, *EIF3 ACTIN* and *RH8*) (Supplementary Fig. S8) were found to be suitable for leaf samples. All of these reference genes were used in the present study for gene expression analyses according to the manufacturer’s instruction (CFx384TM Real-Time System, Bio-Rad, USA) and previous research utilizing multiple reference genes as relative internal controls^78–80^. The coefficient of variation value (CV) for the five control genes was lower than 0.2 (CV = 0.1894), and the average expression stability value (represented by *M*) of the five control genes was lower than 0.5 (*M* = 0.4627), which were used as two default settings^81^. The relative expression level was calculated by the 2^−ΔΔCT^ method with modified amplification efficiency^82^. The expression level was then analyzed with CFX Manager Software version 3.0 (Bio-Rad) using multiple reference genes (*UBC2a*, *YLS8*, *eIF3*, *ACTIN* and *RH8*). Three biological replicates were assessed by the qRT-PCR test, and each sample was tested in triplicate.

### Statistical Analysis

Statistical analysis was performed with SPSS Statistics 17.0 by analysis of variance (ANOVA) based on a t-test (two group comparisons between treated samples and control samples) described in the previous study^63^. Means were considered significantly different based on t-test threshold value corresponding to the *P* value (*P* < 0.05 and *P* < 0.01), which are indicated by asterisks (*) when T_0.975_ (4) > 2.7764, or indicated by (**) when T_0.990_ (4) > 3.7469.

### Data availability

The data sets with respect to the results of this research are deposited in NCBI’s Gene Expression Omnibus (GEO) repository with the GEO Series accession number GSE67559, including the raw sequenced reads generated by the Illumina HiSeq. 2000 and the assembled unique sequences. The hyperlink to these data sets is in http://www.ncbi.nlm.nih.gov/geo/query/acc.cgi?acc = GSE67559.

## Electronic supplementary material

Supplementary Information

Supplementary Dataset 1
