# Supplementary material for: Comparative transcriptome analysis reveals phytohormone signalings, heat shock module and ROS scavenger mediate the cold-tolerance of rubber tree
Source: Sci Rep. 2018 Mar 21;8:4931. doi: 10.1038/s41598-018-23094-y (PMC5862945; doi:10.1038/s41598-018-23094-y)
Supplement: Supplementary file 1 — Supplementary Information [file 41598_2018_23094_MOESM1_ESM.pdf]

**Supplementary Information**

# Comparative transcriptome analysis reveals phytohormone signalings, heat shock module and ROS scavenger mediate the cold-tolerance of rubber tree

Xiaomin Deng[1†], Jianxiao Wang[2†], Yan Li[1], Shaohua Wu[1], Shuguang Yang[1], Jinquan Chao[1], Yueyi Chen[1], Shixin Zhang[1], Minjing Shi[1] & Weimin Tian[1*]

[1]Ministry of Agriculture Key Laboratory of Biology and Genetic
Resources of Rubber Tree/State Key Laboratory Breeding Base of
Cultivation and Physiology for Tropical Crops, Rubber Research Institute, Chinese Ac
-ademy of Tropical Agricultural Sciences, Danzhou, Hainan,
571737, P. R. China.

[2]College of Landscape and Ecological Engineering, Hebei University of Engineering Handan 056021, Hebei China.

[†]These authors have contributed equally to this work.
*Corresponding author: Weimin Tian
Email: wmtian@163.com

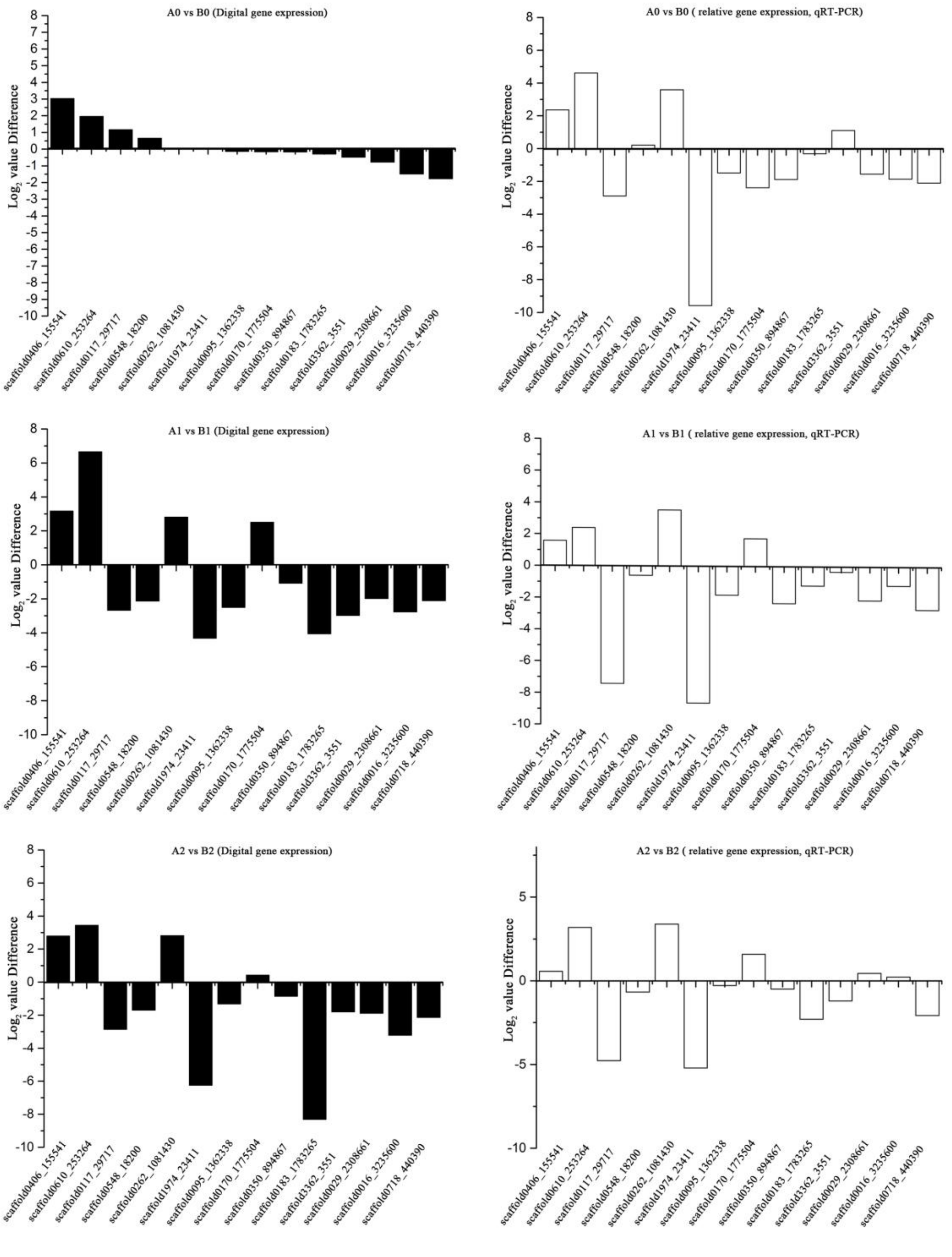


**Figure S1.** Validation of digital gene expression by qRT-PCR. The black column charts show the digital gene expression profiles of the transcriptome. The white column charts show the expression profiles detected by qRT-PCR analysis.

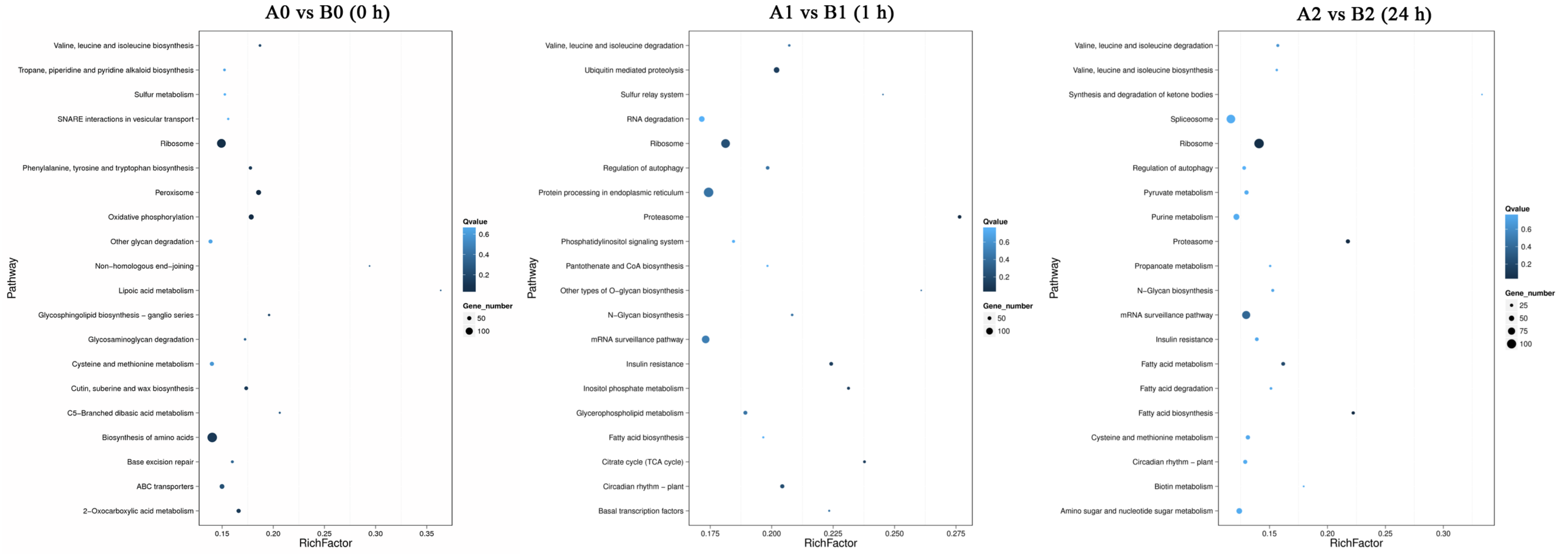


**Figure S2.** Comparison of enriched pathways between '93-114' and 'Reken501' at 0 h, 1 h, and 24 h. The size of the cycles indicates the number of DGEs involved. The density of the color indicates the value of the enrichment factor.

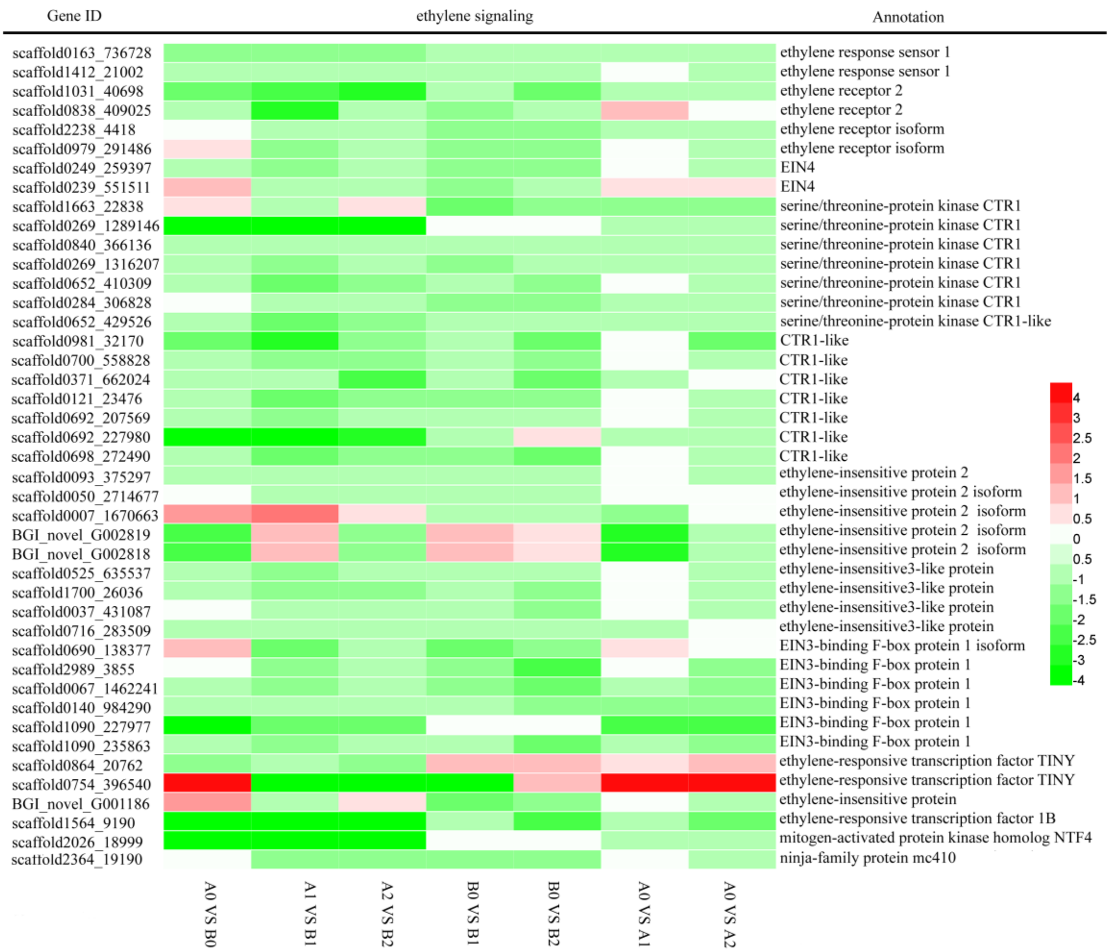


**Figure S3.** Heatmap of the genes related to the ethylene signaling pathway between ‘93-114’ and ‘Reken501’.

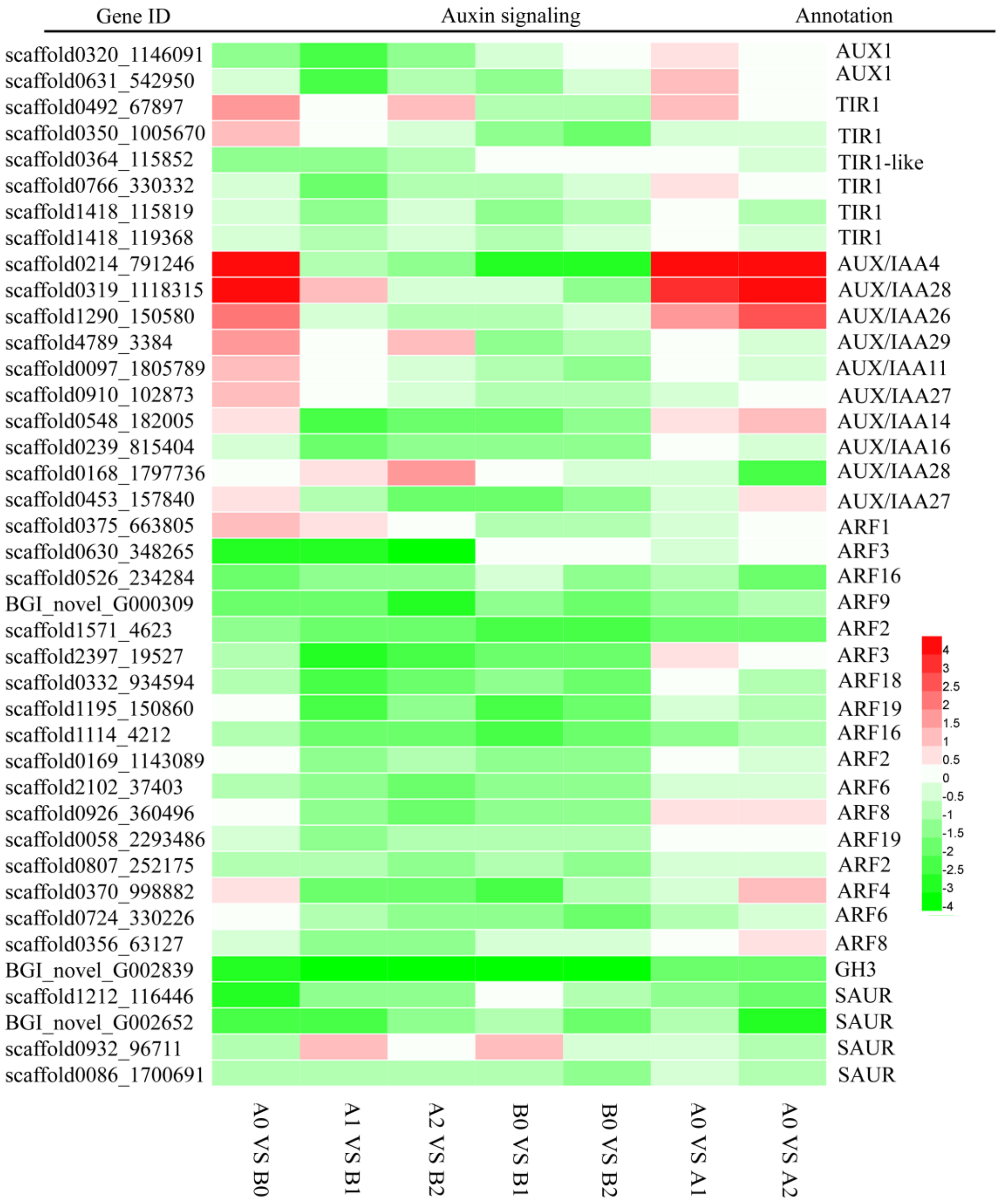


**Figure S4.** Heatmap of the genes related to the auxin signaling pathway between '93-114' and 'Reken501'.

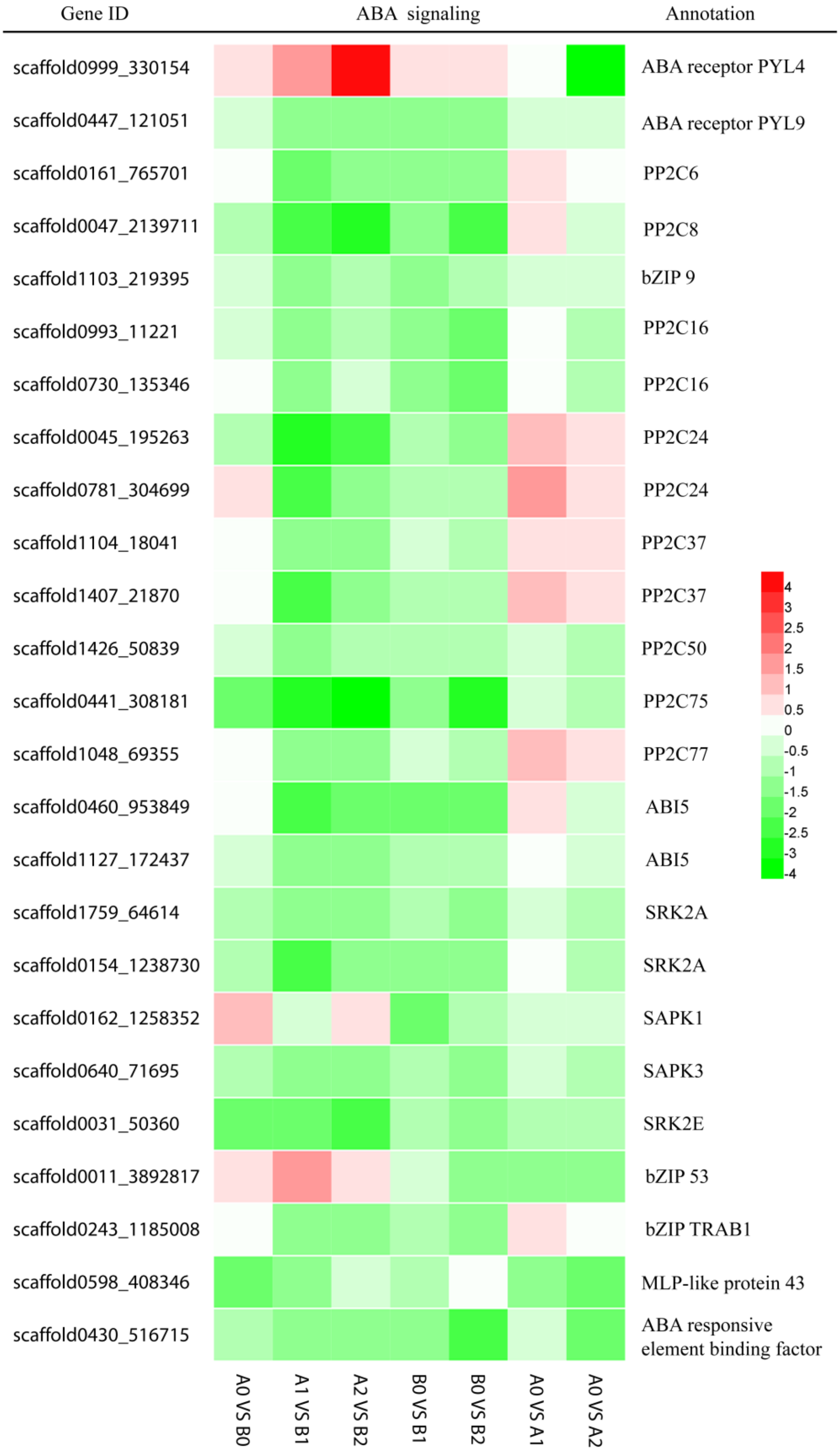


**Figure S5.** Heatmap of the genes related to the ABA signaling pathway between ‘93-114’ and ‘Reken501’.

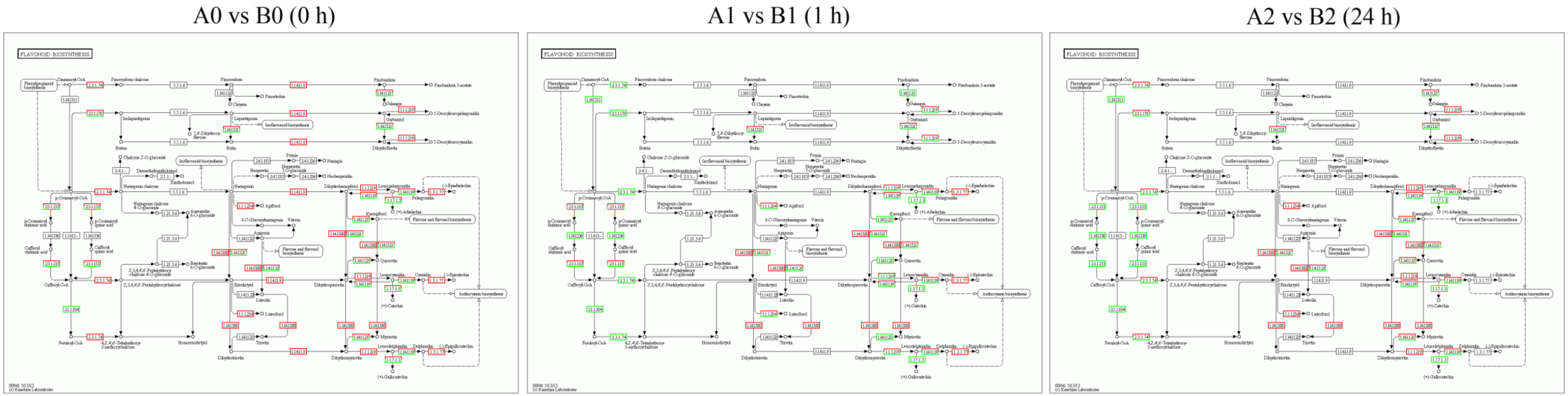


**Figure S6.** Comparison of flavonoid biosynthesis (map00941) analyzed in KEGG database[75-77] between ‘93-114’ and ‘Reken501’ at 0 h, 1 h, and 24 h. The components marked with red and green rectangles are regarded as differentially expressed.

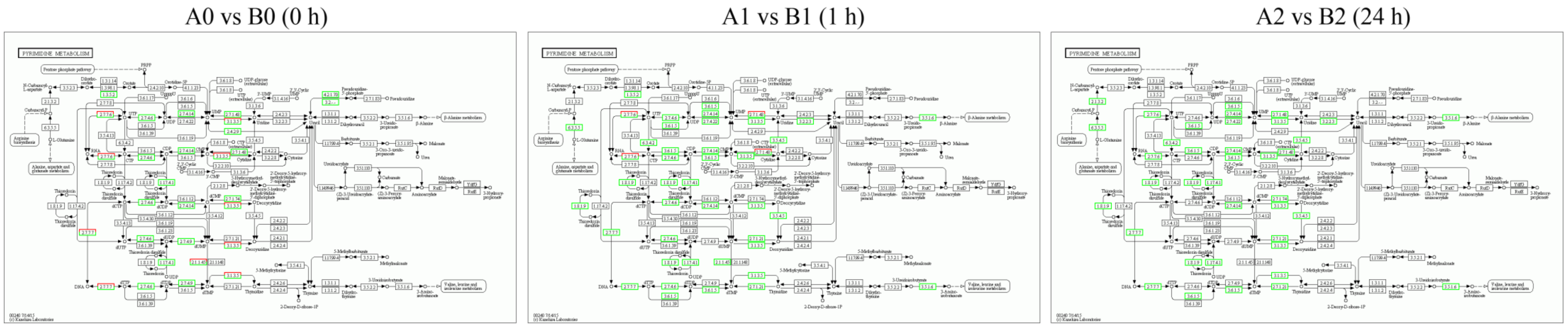


**Figure S7.** Comparison of pyrimidine metabolism (map00240) analyzed in KEGG database[75-77] between '93-114' and 'Reken501' at 0 h, 1 h, and 24 h. The components marked with red and green rectangles are regarded as differentially expressed.

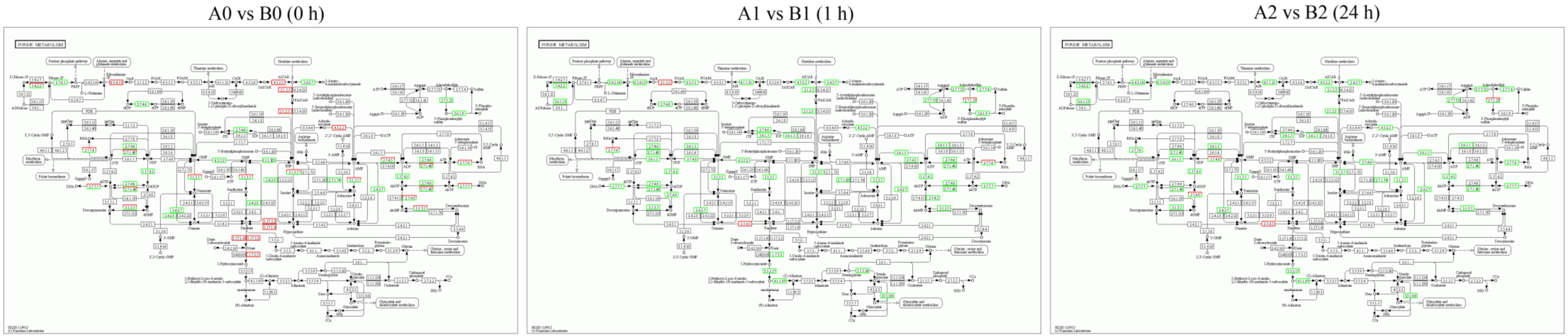


**Figure S8.** Comparison of purine metabolism (map00230) analyzed in KEGG database[75-77] between '93-114' and 'Reken501' at 0 h, 1 h, and 24 h. The components marked with red and green rectangles are regarded as differentially expressed.

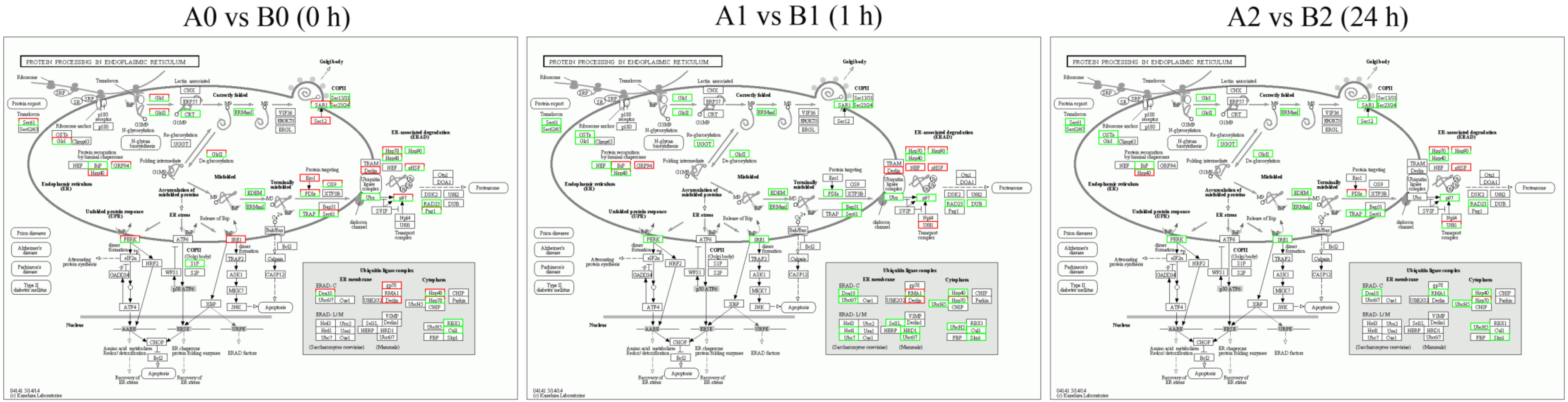


**Figure S9.** Comparison of protein processing in the endoplasmic reticulum (map04141) analyzed in KEGG database[75-77] between ‘93-114’ and ‘Reken501’ at 0 h, 1 h, and 24 h. The components marked with red and green rectangles are regarded as differentially expressed.

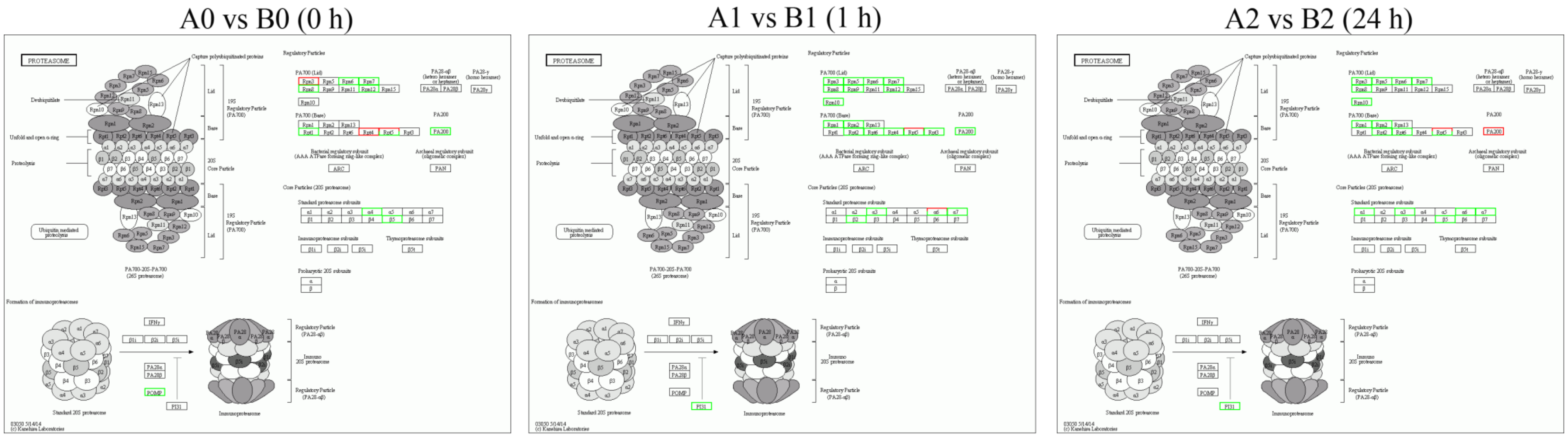


**Figure S10.** Comparison of the proteasome (map03050) analyzed in KEGG database[75-77] between '93-114' and 'Reken501' at 0 h, 1 h, and 24 h. The components marked with red and green rectangles are regarded as differentially expressed.

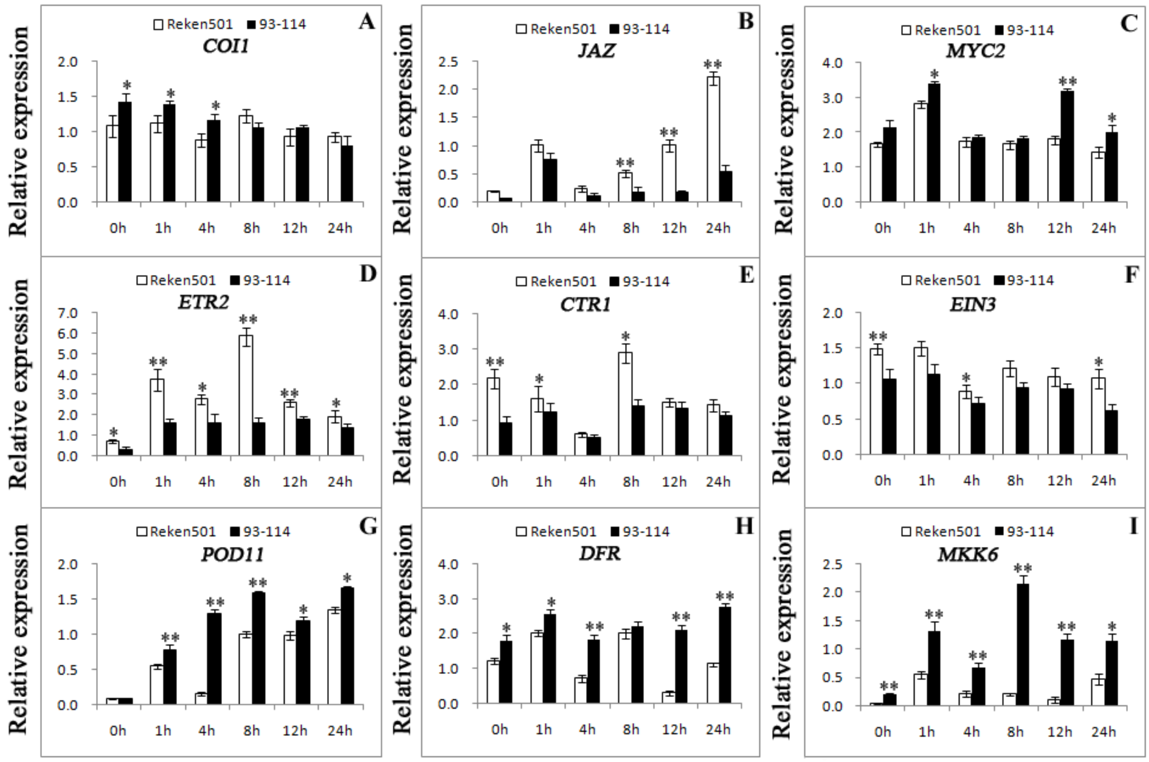


**Figure S11.** The qRT-PCR analysis of stress-related genes in the upstream analysis at different time intervals before and after cold treatment. The relative expression of each gene was calculated as the $2^{-\Delta\Delta Ct}$ value and normalized to the endogenous reference genes. The SD of three biological replicates is indicated by 1 or 2 asterisks depending on the *P* value for the significant difference ($P < 0.05$, $P < 0.01$, respectively) after *t*-test analysis (two group comparisons).

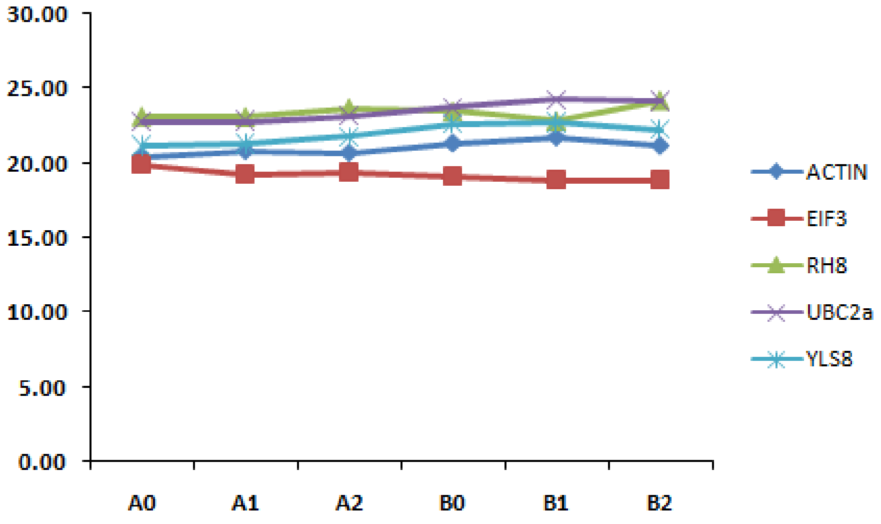


**Figure S12** Evolution of the Cp value for the five reference genes using qRT-PCR analysis. Six samples belong to 'Reken501' (A0, A1 and A2) and '93-114' (B0, B1 and B2). Means were calculated using three biological replicates.

**Table S1.** Primers used in this study for qRT-PCR analysis.

| Gene ID | Forward primer | Reverse primer | Annotation |
|---|---|---|---|
| scaffold0957_116800 | TGGTTAATCAGGTCGGAGAGGA | TCTATGCGGCAACTATCCAAGG | coronatine-insensitive 1 |
| scaffold0609_18761 | AGGTTAAGGAGGTCATGCTCTT | GGAGGAATTGAACTGGTGGATT | plastid jasmonates ZIM-domain protein |
| scaffold0103_1288299 | GGAGGTATTACGAAGCCAAGGA | ATTCCAAGCCACGGATCACTT | transcription factor EMB1444 isoform/MYC2-like |
| scaffold0407_253102 | GCCGTGGCAGACAAGAGTT | AAGTTGGGAAAGCGGGTTCT | dehydration-responsive element-binding protein 2F |
| scaffold0923_363448 | TCGAGTGAAGCTAGTAGGGATG | TGCCATGTCCACCAATAACCT | CRT/DRE binding factor 1 |
| scaffold0082_667097 | CTCACGTACATAGATGAAGAAGCAA | GAATGTGGAGGCGAAAGTAGAAG | dehydration-responsive element-binding protein 1B-like |
| scaffold0082_669813 | GTATTCGACACGCCAAGGTT | CTCAGTAGCATCCGCATCAA | dehydration-responsive element-binding protein 1E-like |
| scaffold0744_127631 | CGGAGGATAGTTACGGTGGTG | CATAACAATCCTTGCCCAAAGC | dehydration-responsive element-binding protein 3-like |
| scaffold1031_40698 | ACCTTCAGATGTCCGAGTTGG | CACACCTCTTCATCAGCACTTG | ethylene receptor 2, ETR2 |
| scaffold1276_47774 | GGATGTGAGGAGCAGGATTACT | ACCACCGTCAGTGAACAACA | dehydration-responsive element-binding protein 2C |
| scaffold0981_32170 | ATTACTTGATGGCAGCAGCAAT | TCCACCTATGATAGCACCACAA | CTR1-like |
| scaffold1700_26036 | ACCTCCTCTACATAGCTTGTCA | CTATGCACCAAAGCATTTGATC | ethylene-insensitive3/ethylene-insensitive3-like protein, EIN3 |
| scaffold0117_29717 | CCAACCACCGTAACTGGAAGT | GCCATCATCACCAACATTGACA | Cu/Zn superoxide dismutase |
| scaffold0718_440390 | GCCCTTGGAGACACAACAAAC | CAGATCACCAGCATGACGATTC | Cu/Zn superoxide dismutase |
| scaffold0195_290440 | ATATACCAACCGCAGATGAAGG | GCACACCGAGCCATTCCTA | peroxidase 11 |
| scaffold3362_3551 | TACACGGATAAGAGAACAAGGG | GGCAGAGCAATTAGCACGAAT | peroxidase 12-like |
| scaffold0823_94491 | TCGTCACTGTTCATCCATCTGT | AATGCACTACTCGTCCTCTAGC | bifunctional dihydroflavonol 4-reductase/flavanone 4-reductase, DFR |
| scaffold0043_1764659 | TAACAGCAAGTCCGAGTGTG | ACCCAAATTGTCCACGCTAC | heat shock transcription factor |
| scaffold0350_894867 | GGTTATGAGGAGCCTGAGACTG | ACCATCCACAGAGCTTGATTCA | heat shock factor protein HSF8 |
| scaffold0985_240417 | GTCAGTCATCCATGCAATGC | CCAGCGAAGTTAGGAAGAGC | heat stress transcription factor A-1b-like |
| scaffold0262_1081430 | CAAACTTCAAGCCCGCAACT | GAACCAGGAGCCTCTGTCAG | heat stress transcription factor A-4a-like |
| scaffold0029_2308661 | AACTGGCGGATGGCTGAA | TCATTTGTGGTGGCTGGTAC | heat stress transcription factor A-8 |
| scaffold0118_132983 | GGTGAGGAGGAGGAAGAGGA | TTCCATCCAACGGCTCTGAT | heat stress transcription factor B-2b |

| | | | |
|---|---|---|---|
| scaffold0015_889453 | CGAAGAGGGTGAGGAGGAAG | ATGGCAGACACATTGGCATT | 17.4 kDa class III heat shock protein |
| scaffold0045_1329 | TGCTTCAAATCAGTGGAGAGAG | AGCCTGAACCTCCTCAATAACT | 17.3 kDa class I heat shock protein |
| scaffold5093_644 | TGGAGCGTAGCAAGGGTAAG | GAACTGTCACCGTCAGAACC | 17.3 kDa class I heat shock protein |
| scaffold1974_23411 | CCAGCAGCCCATGTCTTCA | CATTCCAAGCGATGCCAAGT | 18.1 kDa class I heat shock protein |
| scaffold0530_30694 | TTGGCTCAGGCAGACAAGG | AGTTTCCGTTGGGCATTTGG | Heat shock 70 kDa protein like |
| scaffold0280_1361025 | GGACCTGGTGCTGTTGCTT | CTTCTACGCCAGCCTCATCC | heat shock protein 83-like |
| scaffold0280_1360698 | GCTTGCCGATTTGCTTAGGT | GCTTTCTTGCTCTCACCAGT | heat shock protein 83-like |
| scaffold0548_182005 | GGAGCCCAGGGAATGATAGAC | CACGAGCATCCAGTCACCAT | auxin-responsive protein IAA |
| scaffold0016_3235600 | AAGATGGCATTTCGTTTGGT | TCTACTACAACAGCAGGTGA | DNA-directed RNA polymerase I subunit RPA43 |
| scaffold0095_1362338 | GGAAGCAGGCGGTTTAAGTG | GAGTCGCTCCAGCTCGTATT | dihydroorotate dehydrogenase |
| scaffold0183_1783265 | TTGCAGGTTGATGCTGGAA | TGGTTGGTGCTTGATCTTCA | UMP-CMP kinase |
| scaffold0170_1775504 | TTGCGAGCACATTGAAGGAA | ACAGACCTCTTCCACCTCCT | heat shock 70kDa protein 5 |
| scaffold0610_253264 | GGAACAGGAACCGTGGCTAA | TGCATCGGAAGGAGGAATCT | Trans-resveratrol di-O-methyltransferase like |
| scaffold0406_155541 | CAAGCGTGCTGGTGAAGAG | AAACCCTTTGCGTTGTGGAA | Non-functional NADPH-dependent codeinone reductase 2 like |
| MPKK6 | TGGATCTTGCCTTCATATTCAAAGA | TGCCTACTTCCAAAACCAATGT | Mitogen-activated protein kinase kinase 6 |
| ACTIN | AGTGTGATGTGGATATCAGG | GGGATGCAAGGATAGATC | Reference gene, Actin |
| YLS8 | CCTCGTCGTCATCCGATTC | CAGGCACCTCAGTGATGTC | Reference gene, Mitosis protein YLS8 |
| RH8 | TCACAGGGTTGGTAGATCAG | CCAAGCTCTTGCTCAATCC | Reference gene, DEAD box RNA helicase,RH8 |
| eIF3 | CTGCACAGTAGTCAAGCTCTTTC | CGAAACCCAGATTCCTTCTACCT | Reference gene, Eukaryotic translation initiation factor |
| UBC2a | CATTTATGCGGATGGAAGCA | CAGGGGAGTTTGGATTTGGA | Reference gene, Ubiquitin-protein ligase |

**Supplementary Table S2. The enriched GO terms (Bonferroni-correction P ⩽ 0.05) for DEGs in the comparisons between '93-114' and 'Reken501' at 0 h,1 h, and 24 h.**

| Time | Ontology | Description | Cluster frequency | P value |
|---|---|---|---|---|
| 0 h | molecular_function | Anion binding | 273/1670 | 5.74e-06 |
| | molecular_function | Nucleoside binding | 225/1392 | 0.00024 |
| | molecular_function | Ribonucleoside binding | 225/1384 | 0.00015 |
| | biological_process | phosphorylation | 158/798 | 4.63e-07 |
| | biological_process | oxidation-reduction process | 155/881 | 0.00087 |
| | biological_process | organonitrogen compound metabolic process | 94/484 | 0.00067 |
| 1 h | molecular_function | Anion binding | 583/1670 | 1e-20 |
| | molecular_function | Nucleoside binding | 496/1392 | 2.11e-19 |
| | molecular_function | purine ribonucleoside binding | 492/1375 | 1.17e-19 |
| | biological_process | phosphorylation | 342/798 | 1e-20 |
| | biological_process | regulation of primary metabolic process | 247/614 | 7.51e-13 |
| | biological_process | cellular macromolecule metabolic process | 1234/4172 | 3.18e-07 |
| 24 h | molecular_function | Anion binding | 429/1670 | 1.43e-11 |
| | molecular_function | Nucleoside binding | 362/1392 | 4.06e-10 |
| | molecular_function | Ribonucleoside binding | 362/1384 | 1.73e-10 |
| | biological_process | regulation of macromolecule metabolic process | 191/596 | 6.18e-11 |
| | biological_process | regulation of transcription, DNA-templated | 143/426 | 6.46e-10 |
| | biological_process | regulation of biosynthetic process | 151/469 | 5.87e-09 |
